# miR-150 regulates obesity-associated insulin resistance by controlling B cell functions

**DOI:** 10.1038/srep20176

**Published:** 2016-02-01

**Authors:** Wei Ying, Alexander Tseng, Richard Cheng-An Chang, Haiqing Wang, Yu-lieh Lin, Srikanth Kanameni, Tyler Brehm, Andrew Morin, Benjamin Jones, Taylor Splawn, Michael Criscitiello, Michael C. Golding, Fuller W. Bazer, Stephen Safe, Beiyan Zhou

**Affiliations:** 1 Research Center for Translational Medicine, East Hospital, Tongji University School of Medicine, Shanghai 200120, China; 2 College of Medicine, Texas A&M Health Science Center, College Station, TX, USA; 3 Department of Veterinary Physiology & Pharmacology, College of Veterinary Medicine & Biomedical Sciences, Texas A&M University, College Station, TX, USA; 4 Department of Chemical Engineering, Texas A&M University, College Station, TX, USA; 5 Department of Biochemistry and Biophysics, Texas A&M University, College Station, TX, USA; 6 Department of Agriculture and Life Sciences, Texas A&M University, College Station, TX, USA; 7 Department of Veterinary Pathobiology, College of Veterinary Medicine & Biomedical Sciences, Texas A&M University, College Station, TX, USA; 8 Department of Immunology, University of Connecticut Health Center, Farmington, CT, USA

## Abstract

Adipose tissue resident B cells account for more than 20% of stromal cells within visceral adipose tissues; however, their functions in the adipose tissue niche are poorly elucidated. Here we report that miR-150 modulates adipose tissue function by controlling activation of B cells and their interactions with other immune cells. miR-150KO mice displayed exacerbated obesity-associated tissue inflammation and systemic insulin resistance, which is recapitulated by adoptive transfer of B cells, but not purified immunoglobulin, into obese B^null^ mice. Using purified cell populations, we found that enhanced proinflammatory activation of adipose tissue T cells and macrophages was due to miR-150KO B cells action but not cell-autologous mechanisms. miR-150KO B cells displayed significantly enhanced antigen presentation upon stimulation, ultimately leading to elevated inflammation and insulin resistance, compared to wild type B cells. Knockdown of identified miR-150 target genes, *Elk1*, *Etf1* or *Myb* attenuated B cell action by altering B cell receptor pathways and MHCII cell surface presentation. Our results demonstrate a critical role for miR-150 in regulating B cell functions in adipose tissue which ultimately regulate both metabolic and immunologic homeostasis in the adipose tissue niche.

Metainflammation and insulin resistance are two hallmarks of obesity which contribute to the pathogenesis of obesity-associated diseases, including type 2 diabetes and cardiovascular diseases^1^,^2^,^3^,^4^. Expansion of visceral adipose tissue (VAT) is central to the development of obesity associated metabolic syndromes, characterized by adipocyte malfunction and altered tissue specific immune cell profiles^1^,^3^. Adipose tissue immune cells vary in number and their responses to obese stress^5^. To control the detrimental effects of obesity, it is important to understand the regulatory networks controlling adipose tissue immune cell activation and their interactions within the tissue niche.

The complex immune profile within visceral adipose stroma (VSC) consists of various dynamically interacting cell types which are central to adipose tissue metabolic and immunologic homeostasis. Among VSC immune cells, adipose tissue macrophages (ATMs) account for 30–40% of VSC and the regulation of their activation has been extensively studied^6^,^7^. ATMs display a wide-range of activation statuses from alternative activation (M2) in lean tissue to the predominantly classical pro-inflammatory state (M1) in obese tissues^6^,^7^,^8^. Previous research, including our own, has revealed several key regulators controlling ATM polarization, including nuclear factor κB/c-Jun N-terminal kinase (NFκB/JNK), peroxisome proliferator-activated receptor γ (PPARγ), and microRNAs^9^,^10^,^11^,^12^,^13^. In addition, adipose tissue T cells (ATTs) comprise approximately 10% of obese VSCs and fine-tune the adipose tissue immune environment through direct cell-cell interactions and cytokine production^14^,^15^,^16^. For example, CD8+ T cells secreting interferon γ (IFNγ) promote macrophage infiltration into the adipose tissue, leading to inflammation and subsequent insulin resistance^15^. The proportion of regulatory T (Treg) cells is often decreased in adipose tissue of obese individuals which also facilitates tissue inflammation^14^,^17^. Unlike the other VSC immune cell populations, adipose tissue B cells (ATBs), which represent over 20% of VSCs in obese individuals^18^,^19^, are poorly understood.

ATBs dramatically increase in both absolute number and relative proportion of visceral stromal cells during the development of obesity^18^,^19^. In mouse models of obesity, the accumulation of B cells in visceral adipose tissues peaks 3–4 weeks after initiating high-fat diet (HFD)^19^. ATBs serve as crucial antigen presenting cells within adipose tissue. Mice with defects in B cell formation display significantly lower obesity-induced insulin resistance accompanied with reduced antibody production and perturbed cell-cell interactions^18^,^19^. The regulatory mechanisms modulating ATB response in the face of obesity are yet to be uncovered.

Our previous studies identified microRNAs as crucial regulators controlling ATM polarization and B cell formation^13^,^20^,^21^. miR-150 has been identified as a crucial regulator of B cell formation and function^20^,^21^,^22^. Ectopic expression of miR-150 in hematopoietic stem cells resulted in impaired B cell production by blocking transition from the pro-B to pre-B cell stage without detectable effects on other hematopoietic lineages^21^. In contrast, miR-150 deficiency in mice didn’t significantly alter formation of blood cell lineages derived from hematopoietic stem cells^20^. Furthermore, miR-150KO mice exhibited increased antibody production in the face of antigen challenge^20^. Several target genes of miR-150, including *Myb* (v-myb avian myeloblastosis viral oncogene homolog), *Cbl* (cbl proto-oncogene, E3 ubiquitin protein ligase), *Egr2* (early growth response 2), *Gab1* (GRB2-associated binding protein 1), and *Foxp1* (forkhead box P1^20^,^22^,^23^, are important for B cell formation and function through their effect on various pathways. However, none of these pathways have been explored in the context of ATBs and obesity.

In this study, we show for the first time that miR-150 regulates obesity-induced metainflammation and insulin resistance by controlling ATB function. Using various mouse models, including miR-150KO mice and wild type mice with adoptive transplantation of B cells or antibodies isolated from obese mice, we demonstrate that miR-150 controls activation of ATBs by enhancing the B cell receptor (BCR)-mediated pathways and antigen presentation which is partially mediated by the *Myb*, *Etf1* (eukaryotic translation termination factor 1) and *Elk1* (ETS domain-containing protein) genes. Our results suggest miR-150KO ATBs primarily act through cell-cell interactions, as opposed to pathogenic antibody production, to promote T cell and macrophage activation, resulting in metainflammation and systemic insulin resistance. Our study provides novel insight into microRNA’s regulation of ATBs in an obese context which might identify potential drug targets to mitigate obesity-induced metabolic syndromes.

## Results

### miR-150 deficiency exacerbates obesity-induced metainflammation and systemic insulin resistance

**Figure 1:**
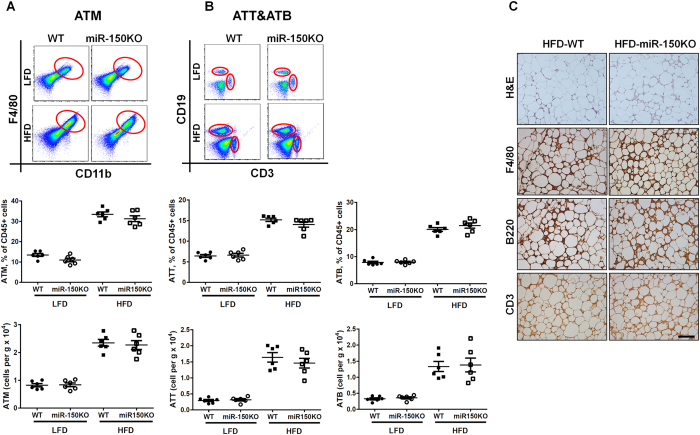
The recruitment of immune cells is dramatically increased during obesity. The population of adipose tissue macrophages (**A**; ATM), adipose tissue B cells (**B**; ATB), and adipose tissue T cells (**C**; ATT) in visceral stromal cells (VSC) of visceral adipose tissues (VAT) of wild type (WT) or miR-150KO mice with or without high-fat diet (HFD). n = 7–10 (**D**) Immune cell components in VAT of HFD-fed WT and miR-150KO mice detected by immunostaining analysis. Scale bar, 100 μm. Data are presented as mean ± SEM.

We first investigated the cell composition in VSCs of lean and diet-induced obese mice. As expected, adipose tissue immune cells were significantly increased in both proportion and absolute cell numbers in the visceral adipose tissue (Fig. 1). ATMs increased from 15% to 35% of VAT immune cells (0.83 ± 0.15 × 10^4 ^cells/g in lean VAT to 2.34 ± 0.30 × 10^4 ^cells/g in obese VAT) (Fig. 1A), and CD3+ ATTs increased from 6% (0.29 ± 0.08 × 10^4 ^cells/g of lean VAT) to 15% (1.6 ± 0.36 × 10^4 ^cells/g of obese VAT) (Fig. 1A,B). Notably, ATB numbers also significantly increased from 8% (0.33 ± 0.09 × 10^4 ^cells/g of VAT) in lean to 20% of immune cells (1.22 ± 0.30 × 10^4 ^cells/g of VAT) in obese mice (Fig. 1C). Surprisingly, mice with miR-150 depletion (miR-150KO) did not exhibit significantly different proportions of ATMs, ATTs, or ATBs as determined by flow cytometry and immunostaining analysis (Fig. 1A–D). Obese WT and miR-150KO mice exhibited similar circulating or splenic immune cell populations compared to each other and their lean counterparts (Supplementary Figures S1 and S2), including total B cells (CD19+), T cells (CD3+), neutrophils (CD11b + Gr1+) and monocytes (CD11b + Gr1-F4/80). Therefore, it is unlikely that elevated populations of immune cells in VAT are simply a result of enhanced hematopoiesis.

**Figure 2:**
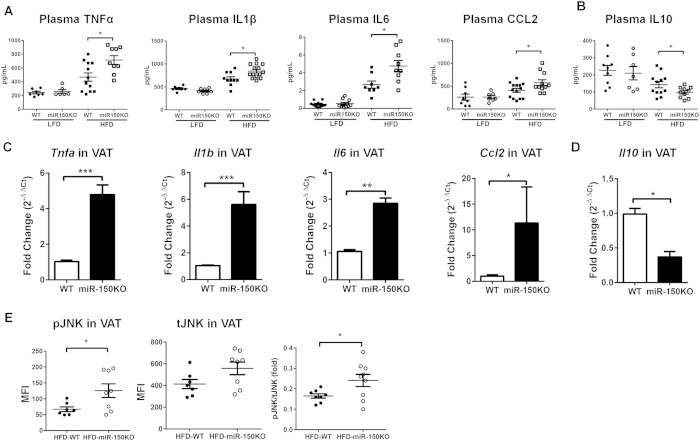
miR-150 regulates obesity-induced inflammation. (**A,B**) Concentrations of cytokines in plasma of wild type or miR-150KO mice with or without HFD. n = 7–10. (**C**) The expression of inflammatory cytokines tumor necrosis factor-α (TNFα), interleukin (IL) 1β, IL6, and chemokine (C-C motif) ligand 2 (CCL2) in VAT of HFD-fed mice. n = 3. (**D**) The expression of IL10 in VAT of HFD-fed mice. n = 3. (**E**) Activation of c-Jun N-terminal kinases (JNK) signaling pathway in VAT of mice on a HFD. MFI, medium fluorescence intensity. n = 7–8. (**F**) Activation of nuclear factor-κB (NFκB) pathway in VAT of HFD-fed mice. n = 4. Data are presented as mean ± SEM. *P < 0.05, **P < 0.001, ***P < 0.0001, Student’s *t* test.

**Figure 3:**
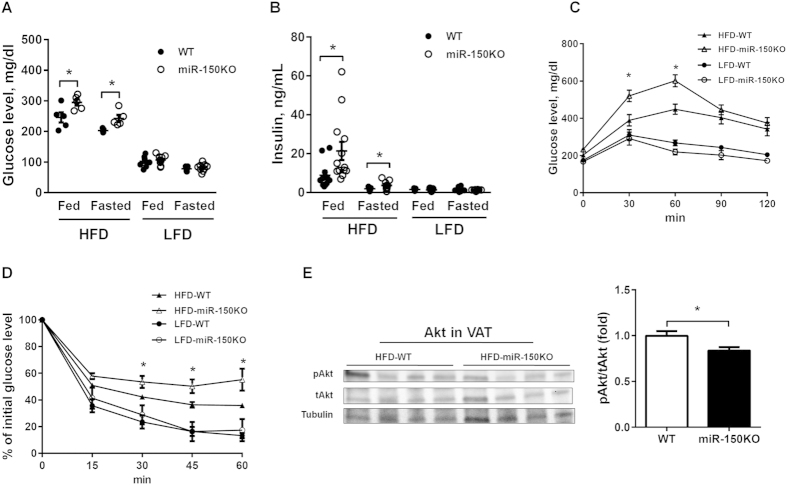
miR-150 ablation exacerbates obesity-induced systemic insulin resistance. (**A,B**) Concentrations of glucose and insulin in plasma of miR-150KO mice or wild type mice fed a HFD or a low-fat diet (LFD), or fasted for 16 hours. n = 6–10. (**C,D**) Glucose tolerance test and insulin tolerance test after 12 weeks of feeding. n = 6–10. (**E**) Activation of Akt signaling pathway in VAT of mice on a HFD 5 minutes following an acute insulin injection. n = 4. Data are presented as mean ± SEM. *P < 0.05, Student’s *t* test.

Despite similar food intake and body weight gain over a 12-week feeding period (Supplementary Figure S3A and B), as well as comparable adiposity (Supplementary Figure S3C), HFD-miR-150KO mice exhibited severely exacerbated obesity-induced metainflammation and insulin resistance (Figs 2 and 3). At a systemic level, concentrations of inflammatory cytokines and chemokines in plasma, including tumor necrosis factor α (TNFα), interleukin (IL) 1β, IL6 and chemokine (C-C motif) ligand 2 (CCL2), were significantly increased (Fig. 2A) in obese wild type mice compared to age-matched lean mice fed a low-fat diet (LFD); whereas concentrations of IL10 in plasma (Fig. 2B) were significantly lower in obese mice. The overall proinflammatory pattern of cytokines in plasma was even greater in miR-150KO mice fed a HFD (Fig. 2A,B). For example, expression of TNFα, IL1β, IL6 and CCL2 was significantly higher in the visceral fat pads from HFD-miR-150KO mice than from HFD-WT mice (Fig. 2C,D). The elevated inflammatory milieu in HFD-miR-150KO mice was further confirmed by enhanced activation of JNK pathway in visceral white fat (Fig. 2E).

MiR-150 null mice also developed severe obesity-induced hyperglycemia and hyperinsulinemia. Under both fed and fasted conditions, HFD-miR150KO mice exhibited significantly higher plasma levels of glucose (Fig. 3A) and insulin (Fig. 3B) than HFD-WT mice; whereas both glucose and insulin levels were similar in mutant and wild type mice on chow diet (Fig. 3A,B). These results suggest that the impact of miR-150 deletion is unmasked by HFD-induced obesity. Indeed, when challenged with a single dose of glucose (2 g/kg) or insulin (1 U/kg), miR-150KO mice on HFD, but not on LFD, exhibited severe glucose intolerance (Fig. 3C) and insulin resistance (Fig. 3D) compared to wild type mice. Furthermore, analysis of Akt activation in VAT and liver after portal vein insulin injection indicated that miR-150 deficiency significantly impaired the insulin signaling pathway in mice under obese stress. Although Akt activation in the skeletal muscle did not reach statistical significance, it did display a consistent trend towards decreased activation in miR-150KO animals (Fig. 3E, Supplementary Figure S4). Taken together, these results suggest that miR-150 is a crucial regulator of metabolic function in adipose tissue of mice with diet-induced obesity.

### miR-150 deficiency directly enhances adipose tissue B cell function

In addition to the dysregulated metabolic phenotype observed in miR-150KO mice, our analysis of individual immune compartments in VAT revealed that the enhanced ATB activation profile in obesity was further exacerbated by miR-150 deficiency (Supplementary Figure S5). Furthermore, obese miR150KO ATMs displayed a more proinflammatory phenotype compared to WT obese ATMs as demonstrated by increased proportions of proinflammatory CD206-CD11c+ M1 macrophages and significantly enhanced proinflammatory gene expression (Supplementary Figure S6). We did not observe similar differences in CD4+ and CD8+ ATT activation profiles (Supplementary Figure S5).

**Figure 4:**
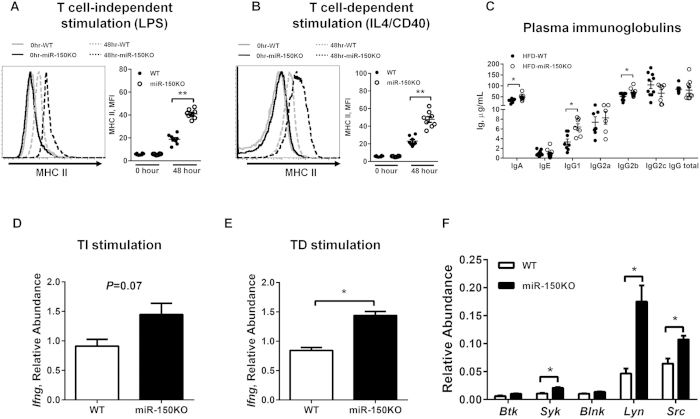
miR-150 is an important regulator of adipose tissue B cell function. (**A,B**) The production of MHC II by splenic B cells after stimulation with lipopolysaccharide (LPS, 10 μg/mL) or IL4 (10 ng/mL)/anti-CD40 (10 μg/mL). n = 9. (**C**) The concentration of immunoglobulins (Ig) in plasma of mice on a HFD. n = 7–10. (**D,E**) The gene expression of interferon (IFN) γ by B cells after 72 hours LPS or IL4/anti-CD40 stimulation. n = 4. (**F**) The gene expression of B cell receptor (BCR) signaling pathway components in WT or miR-150KO B cells. n = 3. Splenic B cells were derived from 6–8 week old WT or miR-150KO chow diet fed mice. Data are presented as mean ± SEM. *P < 0.05, **P < 0.001, Student’s *t* test.

Next, we determined whether loss of miR-150 alters B cell, T cell or macrophage activation directly or indirectly using two sets of experiments: (a) purified cell populations subjected to activation; or (b) co-culture analysis to examine cell-cell interaction mediated activation. We found that loss of miR-150KO did not significantly change the activation profiles of macrophages with respect to polarized or acute phase stimulation (Supplementary Figures S7 and S8). Similarly, isolated miR-150KO CD4+ or CD8+ T cells responded to stimuli identically to wild type T cells with respect to surface marker induction and cytokine production (Supplementary Figures S7 and S9). Interestingly, isolated miR-150KO B220+ B cells displayed significantly enhanced activation profiles compared to wild type B cells when subjected to either LPS (T cell-independent, TI) or IL4/anti-CD40 (T cell-dependent, TD) (Fig. 4A), although PMA/ionomycin short-term treatment did not alter the activation profiles (Supplementary Figure S7). Compared to wild type B cells, we observed a dramatic increase in the proportion of miR-150KO B cells that displayed major histocompatibility complex II (MHCII) on the cell membrane at 72 hours post stimulation with TI stimuli (Fig. 4A). miR-150KO B cells also exhibited higher membrane levels of MHCII complex than wild type B cells exposed to TD stimuli (Fig. 4B). These findings are consistent with the elevated immunoglobulin levels in plasma from HFD-miR-150KO mice compared to wild type mice (Fig. 4C). Higher levels of IgA and IgG subtypes IgG1 and IgG2b, and enhanced interferon γ (IFNγ) expression post stimulation, were observed in plasma of HFD-miR150KO mice (Fig. 4C,D,E). Given the essential role of BCR signaling in initiating B cell responses^24^,^25^, we further evaluated gene expression of BCR-mediated signaling pathway components. Several important components of this pathway were significantly increased, including *Syk* (spleen tyrosine kinase), *Lyn*(v-yes-1 yamaguchi sarcoma viral related oncogene homolog) and *Src* (v-**src** avian sarcoma (Schmidt-Ruppin A-2) viral oncogene homolog), suggesting that enhanced B cell responses due to miR-150 deficiency are likely BCR-dependent (Fig. 4F). In summary, these results suggest that miR-150 deficiency does not directly affect macrophage polarization or T cell activation, but can enhance B cell activity in response to stimuli.

### miR-150KO ATBs are critical contributors to obesity associated glucose intolerance

**Figure 5:**
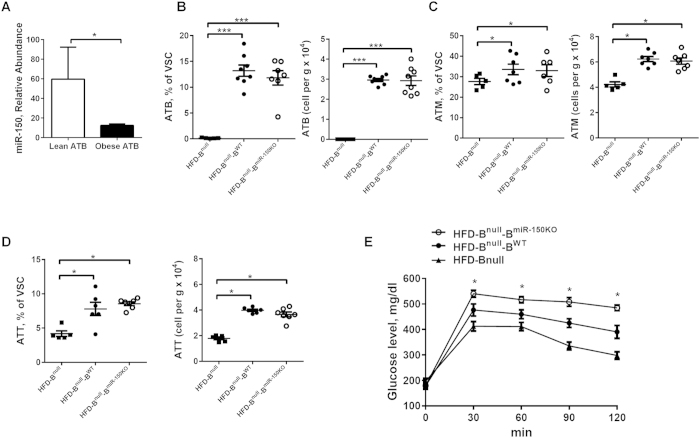
miR-150 deficiency-dependent insulin resistance is mediated by adipose tissue B cells. (**A**) miR-150 expression in B cells of lean and obese mice. n = 4. (**B–D**) The infiltration of B cells (CD19+), macrophages (F4/80 + CD11b+), and CD3+ T cells in VSC of HFD-B^null^ mice after 2 weeks injection of B cells. n = 5–8. (**E**) Glucose tolerance test of HFD-fed B^null^ mice after 2 weeks injection of B cells. n = 7–8. HFD-B^null^-B^miR-150KO^, HFD-fed B^null^ mice injected with miR-150KO B cells; HFD- B^null^-B^WT^, HFD-fed B^null^ mice injected with WT B cells. Data are presented as mean ± SEM. *P < 0.05, ***P < 0.0001, Student’s *t* test.

We observed dramatically reduced expression of miR-150 in VAT B-cells isolated from obese mice (Fig. 5A). To understand if miR-150 null B cells are major contributors to obesity-associated chronic inflammation and systemic inflammation, we utilized an adoptive transplantation assay. Splenic B cells isolated from obese mice were validated for purity and viability (Supplementary Figure S12) and injected into mice lacking endogenous B cells due to mutations in the expression of IgM (B^null^; Supplementary Figure S13) and fed a HFD for 12 weeks^26^. Body weight gain and adiposity were monitored post transplantation and significance differences were not observed between mice that received 1 × 10^7^ B cells from either HFD-miR-150KO (B^miR-150KO^) or HFD-WT (B^WT^) mice (Supplementary Figure S14). Engraftment of B^miR-150KO^ or B^WT^ cells in the adipose tissue was confirmed using flow cytometric analysis (Fig. 5B). As expected, HFD-B^null^ mice that received WT or miR 150KO B cells displayed a comparable cell composition of ATBs (Fig. 5A), ATMs (Fig. 5C), and ATTs (Fig. 5D) in VSCs. Two weeks after B cell transplantation, recipient mice were subjected to a glucose tolerant test. Consistent with previous observations^19^, B^null^ mice fed a LFD had glucose responses that were indistinguishable from wild type mice; however, after HFD feeding, B^null^ mice exhibited less glucose intolerance compared to HFD-WT mice (Supplementary Figure S15). Following adoptive transplantation, HFD-B^null^ mice that received WT B cells displayed higher glucose intolerance compared to non-B cell transplanted mice (Fig. 5E). Moreover, transplantation of B^miR-150KO^ cells into HFD-B^null^ mice exacerbated the glucose intolerance observed in HFD-B^null^-B^WT^ mice (Fig. 5E). Furthermore, transplantation of obese B^miR-150KO^ cells significantly increased the elevated fasting plasma insulin and glucose seen in HFD-B^null^-B^WT^ mice (Supplementary Figure S15). Transplantation of WT B cells into obese VAT also increased VAT inflammatory gene expression, which was further elevated in B^miR-150KO^ transplanted animals (Supplementary Figure S16). These results suggest that miR-150 deficiency in B cells is sufficient to exacerbate glucose intolerance in obese mice.

### miR-150 regulates B cell-dependent interactions with macrophages and T cells ultimately altering adipose tissue function

Given that B cells are major antigen presenting cells in the adipose tissue niche and the primary source of immunoglobulins^27^,^28^,^29^,^30^, we further investigated if miR-150 regulated B cells by altering their cell-cell interactions or production of immunoglobulins in the adipose tissue niche. HFD-miR-150KO mice exhibited higher amounts of immunoglobulin in plasma than HFD-WT mice (Fig. 4C); therefore we purified immunoglobulins using the NAb^TM^ Protein L Spin kit (Supplementary Figure S17). Purified immunoglobulins were injected into HFD-B^null^ mice, which have no endogenous immunoglobulin production. Body weight gain was monitored followed by a glucose tolerance test at 7 days post injection of antibody. Surprisingly, glucose intolerance was similar in mice receiving purified immunoglobulin from miR-150KO or WT mice (Supplementary Figure S18). Although our results do not totally rule out the importance of total immunoglobulins in controlling obesity-induced glucose intolerance, it is likely that the effects of miR-150 on obesity-induced insulin resistance are not related to production of pathogenic antibodies.

**Figure 6:**
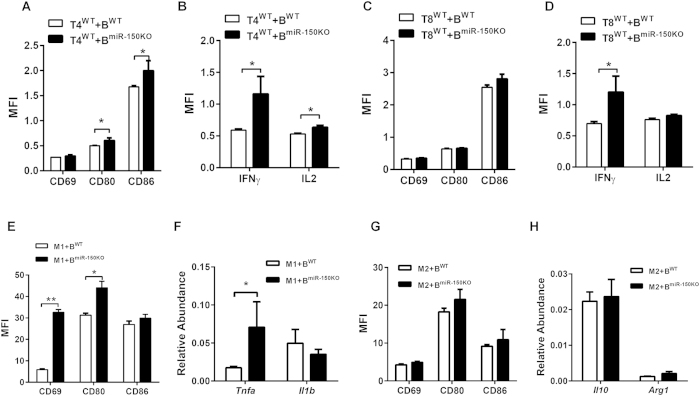
miR-150 may modulate obesity-associated insulin resistance and tissue inflammation by regulating B cell interactions with other immune cells. (**A–D**) The activation of CD4+ T cells (T4) or CD8+ T cells (T8) after co-culture with activated WT or miR-150KO B cells. n = 6. (**E–H**) The expression level of BMDM activation-related surface markers and key genes after 48 hours co-cultured with activated WT or miR-150KO B cells. Splenic CD4+ or CD8+ T cells and B cells were used for co-culture experiments. All T cells, B cells, and BMDMs were derived from 4–6 week old miR-150KO or WT chow diet fed mice. n = 6. Data are presented as mean ± SEM. *P < 0.05, **P < 0.001, Student’s *t* test.

Next, we investigated if miR-150 modulates B-cell interactions with other cell types in the adipose tissue niche. First, we co-cultured purified mature adipocytes from HFD-WT mice with activated WT or miR-150KO B cells. Interestingly, no significant difference was observed in the adipocytes with respect to production of proinflammatory cytokines (Supplementary Figure S19). Although loss of miR-150 did not significantly affect the responses of purified T cells or macrophages to stimuli, it is possible that enhanced activation of ATBs enhances their activities in VSCs from obese individuals through cell-cell interactions. Therefore, we examined activation of T cells and macrophage in the presence of B cells with or without miR-150 deletion. Wild type CD4+ and CD8+ T cells displayed a higher response to CD3-CD28 activation when co-cultured with activated miR-150KO B cells compared to wild type B cells, resulting in higher levels of activation-related surface markers and increased production of IFNγ and IL2 (Fig. 6A–D). Although miR-150 depletion in macrophages did not alter their activation (Supplementary Figure S4), proinflammatory M1 macrophages co-cultured with miR-150KO B cells displayed a significantly enhanced proinflammatory activation profile, including activation-related surface markers and production of cytokines (Fig. 6E,F). M2 cells showed a similar activation status when co-cultured with either WT or miR-150KO B cells (Fig. 6G,H). Taken together, these results suggest that miR-150 potentially modulates obesity induced adipose tissue inflammation through controlling B cell function interactions with macrophages and T cells.

### Several genes targeted by miR-150 mediate its regulation of B cell functions

**Figure 7:**
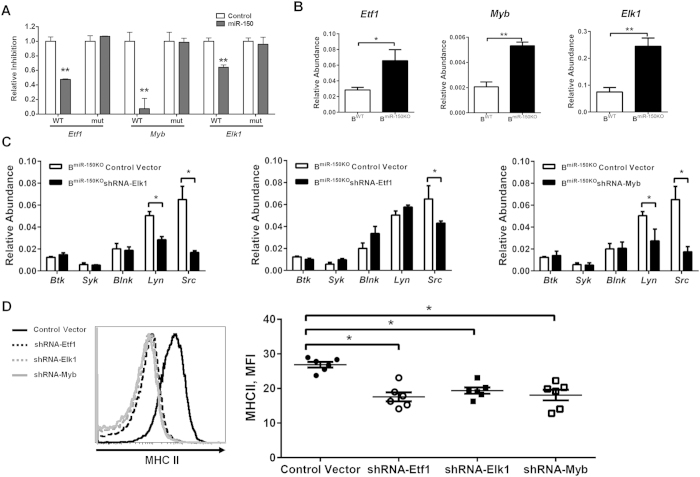
miR-150 controls B cell functions through multiple target genes. (**A**) Reporter constructs containing a 3′ untranslated region (UTR) with WT or mutated miR-150 binding sites of target genes (mut). n = 3. (**B**) After 72-hour activation, the expression of miR-150 target genes in splenic WT or miR-150KO B cells. n = 3. (**C**) The activation of BCR signaling pathways in miR-150KO B cells with knockdown of miR-150 target genes in response to LPS stimulation. n = 3. (**D**) MHC II production by miR-150KO B cells after knockdown of miR-150 target genes in response to LPS stimulation. Splenic B cells were derived from 6–8 week old miR-150KO chow-diet fed mice. n = 6. Data are presented as mean ± SEM. *P < 0.05, **P < 0.001, Student’s *t* test.

MicroRNAs exert their biological functions by either blocking translation and/or inducing degradation of target mRNAs by base-pairing to recognition sites^31^,^32^. *Myb* is reported to be an important target gene of miR-150 during B cell formation and activation^20^,^33^,^34^,^35^,^36^,^37^. Utilizing prediction algorithms, TargetScan Mouse 6.2^38^ and PicTar^39^, we identified several additional genes bearing miR-150 target sites. Using a luciferase reporter assay, we confirmed that *Elk1* and *Etf1* are *bona fide* miR-150 target genes as evidenced by suppression in their luciferase activities in the presence of miR-150 (Fig. 7A). In contrast, mutation of the miR-150 binding site in the 3′ untranslated regions (UTR) of *Elk1* and *Etf1* prevented inhibition of luciferase activity by miR-150 (Fig. 7A). Expression of miR-150 target genes *Elk1*, *Etf1*, and *Myb* was also enhanced in miR-150KO B cells during *in vitro* activation (Fig. 7B). To further evaluate the effects of target genes on B cell functions, we knocked down expression of these genes in isolated B cells with short hairpin RNA (shRNA) constructs. The knockdown efficacy was evaluated in transfected cells by quantitative PCR analysis (Supplementary Figure S20). Intriguingly, knockdown of *Myb*, *Etf1*, or *Elk1* significantly decreased activation of B cells with respect to activation of BCR signaling pathways and MHC II membrane expression, as compared to miR-150KO B cells infected with a control vector harboring scrambled target sites (Fig. 7C,D). These results demonstrated that *Myb*, *Elk1* and *Etf1* are bona fide targets of miR-150, and play critical roles in mediating miR-150’s effects on B cell functions.

## Discussion

Chronic adipose tissue inflammation is a major pathogenic phenotype associated with obesity^1^,^2^,^4^. Dramatic increases in immune cell populations and their enhanced inflammatory capabilities alter adipose tissue functions to exacerbate pre-diabetic conditions, including systemic insulin resistance and inflammation^5^,^15^,^40^,^41^. Within the adipose tissue, B cells contribute more than 20% of the stromal cell population^18^,^19^. However, their overall impact on physiological alterations, both metabolic and inflammatory, and mechanisms underlying their actions in adipose tissues of obese individuals are poorly defined. In this study, we found that miR-150, a hematopoietic enriched microRNA exerting profound regulatory effects on B cell formation and function, is a novel molecule regulating obesity-associated adipose tissue inflammation and insulin resistance.

Previous studies identified several hematopoietic enriched microRNAs including miR-150, which is a critical regulator of B cell formation^20^,^21^. Ectopic expression of miR-150 in all tissues or in hematopoietic stem cells, significantly and specifically impairs B lineage development by blocking the transition from pro-B to pre-B stages. Interestingly, deletion of miR-150 resulted in undetectable changes in hematopoietic lineages and non-hematopoietic tissues, but increased B cell activation in response to stimuli. Indeed, we observed exacerbated pre-diabetic conditions, including elevated levels of insulin and cytokines, in miR-150KO mice compared to wild-type controls fed a HFD. Furthermore, we now confirm that deletion of miR-150 in B cells significantly alters B cell activation in response to both T cell-dependent and T cell-independent stimuli with respect to increasing activation of BCR signaling pathways and immunoglobulin production (Fig. 4). More importantly, miR-150 significantly affects antigen presentation on the B cell membrane, as evidenced by a higher abundance of MHC II on miR-150 null B cells in response to stimuli (Fig. 4).

Our adoptive transplantation model using B^null^ mice demonstrated that the transfer of B cells from HFD-miR150 or HFD-WT mice partially recapitulated the insulin resistance characteristic of miR-150 mutant mice (Fig. 5D). Winer *et al.* suggested that immunoglobulin production by B cells could contribute to insulin resistance^19^; however, we failed to observe such a contribution in response to purified immunoglobulins from HFD-miR-150KO or HFD-WT mice. We observed that B cells lacking miR-150 displayed a slight increase in immunoglobulin production, which could partially contribute to higher circulating levels of IgG and IgA in plasma of mice fed a HFD. However, the specific contribution of immunoglobulins produced by B cells, especially adipose tissue B cells, to the development of insulin resistance needs to be further investigated. Taken together, our results suggest that B cells in adipose tissue are primary contributors to adipose tissue inflammation and systemic insulin resistance in obese miR-150KO mice.

**Figure 8:**
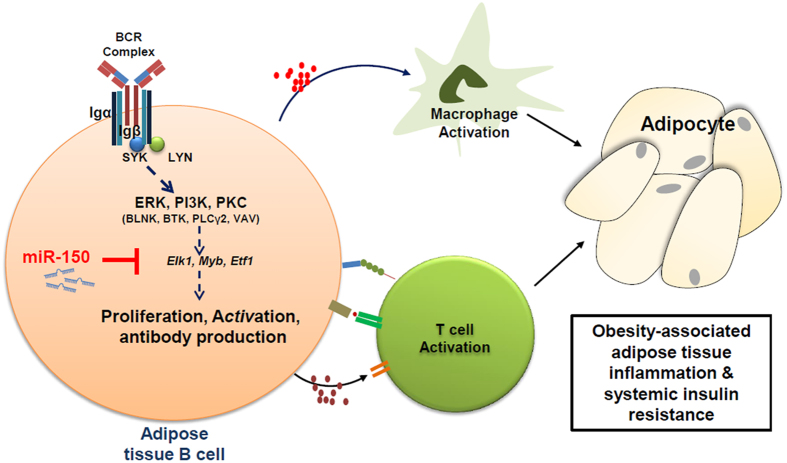
Schematic model of miR-150-mediated molecular network in B cells. 673 miR-150 directly suppresses the expression of target genes, *Elk1*, *Myb*, *Etf1*, which are 674 critical mediators of activation of the B cell receptor (BCR) signaling cascade. This miR-675 150-mediated B cell function is also critical for the activation of T cells and 676 macrophages residing in adipose tissues, eventually exerting profound impacts on the 677 function of adipocytes. *Elk1*, ETS domain-containing protein. *Myb*, myeloblastosis 678 oncogene. *Etf1*, eukaryotic translation termination factor 1. SYK, spleen tyrosine kinase. 679 LYN, tyrosine-protein kinase. ERK, extracellular regulated MAP kinase. PI3K, 680 phosphoinositide 3-kinase. PKC, protein kinase C. BLNK, B-cell linker. BTK, Bruton’s 681 tyrosine kinase. PLCγ2, phospholipase C, gamma 2. VAV, guanine nucleotide 682 exchange factor. Figure contributed by W. Ying.

Although loss of miR-150 did not significantly alter activation states of macrophages and T cells under the obese stress(Supplementary Figures S4 and S5)^21^, the overall proinflammatory profile was enhanced (Fig. 2). We confirmed that miR-150KO B cells can enhance both T cell and macrophage activation profiles (Fig. 6) which can impair insulin tolerance in adipose tissue transplanted with miR-150KO B cells (Fig. 5D). In addition, enhanced T cell and macrophage infiltration into adipose tissue in the adoptive transplantation model suggests that ATBs release cytokines promoting the recruitment of immune cells in obesity. Together with increased T cells and macrophages, adipose tissue B cells exacerbate obesity-induced chronic inflammatory features in the adipose tissue and subsequent systemic insulin resistance (Fig. 8).

Our understanding of the B cell contribution to adipose tissue function, especially under the stress of obesity, is in its infancy. In this study, we demonstrate that miR-150, a B cell enriched regulatory microRNA, plays a role in modulating the pathogenesis of obesity associated adipose tissue inflammation and insulin resistance, which are major contributors to metabolic syndrome and development of type 2 diabetes. The ability of miR-150 to simultaneously target multiple genes allows it to exert a potent impact by altering signaling networks governing adipose tissue B cell function (Fig. 8). These miR-150 target genes, *Myb*, *Elk1*, and *Etf1*, are known to be important in regulating B cell differentiation and function. For example, the transcription factor MYB is required for the expression of IL7 receptor and EBF1 that are essential for the intrinsic survival of pro-B cells^34^. ELK1 is a downstream component of ERK kinases-mediated pathways that are critical for the pre-BCR-regulated early B cell expansion^42^. ETF1 is critical to control the translation of messenger RNAs into protein by its properties as a polypeptide chain release factor^43^. Surprisingly, our results indicated that knockdown of these miR-150 targets reduced expression of upstream BCR signaling pathway components (i.e. src and lyn), suggesting the potential involvement of miR-150 in modulating a feedback circuit in B cell activation. Further investigation regarding the mechanisms of miR-150 and B cell action will greatly facilitate development of therapeutics treating obesity-associated complications by targeting microRNAs and modulating B cells.

## Methods

### Animal Experiments

The generation of miR-150-deficient (miR-150KO) and B^null^ B6 mice has been previously described^21^,^26^. Wild-type (WT) C57BL/6J mice were used as controls. All mice were maintained on a 12/12-hour light-dark cycle. Male mice 5 to 6 weeks of age were fed ad libitum. Mice were fed a high-fat diet (60% fat calories, 20% protein calories, and 20% carbohydrate calories) or a low-fat diet (10% fat calories, 20% protein calories, and 70% carbohydrate calories) for 12 weeks. After the feeding regimen, mice were subjected to phenotype characterization and metabolic assays, including measurement of metabolic parameters in plasma, insulin and glucose tolerance tests.

### Acute Insulin Challenge

We performed portal vein insulin injection to assay the sensitivity of the insulin signaling pathway (Humulin R, Eli Lilly). Following the 12-week feeding period, HFD-fed mice were fasted for 16 hours. Subsequently, mice were anesthetized with inhaled isoflurane, their abdominal cavity opened and portal vein exposed. An insulin bolus (2 U/Kg body weight in 100 ul normal saline) was injected into the portal vein. Within 5 minutes of the injection, liver, skeletal muscle, and VAT were excised and snap frozen in liquid nitrogen. Samples were stored in −80 °C until further processing.

### Purification and transfer of B cells

After 12-week on a HFD, spleens were collected from HFD-fed WT and miR-150KO mice and mechanically dissociated. After lysing red blood cells, single splenic B220+ B cells were purified by using anti-mouse CD45R/B220 conjugated to magnetic particles (Cat. No. 551513, BD Biosciences). B cell purity and B cell subtypes were determined by flow cytometry using an antibody against B220 (Supplementary Figure S12). A total of 1 × 10^7^ B220+ B cells in 150 μL of PBS were transferred into each HFD-fed B^null^ mouse via intraperitoneal (i.p.) injection. HFD B^null^ mice without B cell transfer served as controls. Glucose tolerance was determined 2 weeks after the B cell injection.

### Isolation and transfer of immunoglobulin

Immunoglobulin proteins were isolated from plasma of HFD-WT or miR-150KO mice using NAb^TM^ Protein L Spin kit (Cat. No. 89981, Life Technologies). Immunoglobulin proteins were validated by gel electrophoresis (Supplementary Figure S17). A total of 150 μg of purified proteins in 150 μL of PBS were transferred into each HFD-fed B^null^ mouse via intraperitoneal injection on Day 0 and Day 3. HFD-fed B^null^ mice without immunoglobulin protein transfer served as controls. A glucose tolerance test was performed 1 week after injection of immunoglobulin.

### Isolation of stromal cells from visceral adipose tissues (VATs)

Visceral adipose tissues was mechanically chopped and then digested with collagenase II (Cat. No. 17101, Life Technologies) for 30 min at 37 ^o^C. After lysing red blood cells and passing cells through a 200 μm cell strainer, visceral fat stromal cells were collected following centrifugation at 1000 x g for 5 mins.

### Immunoglobulin concentration measurement

Concentrations of immunoglobulin including IgA, IgE, IgG1, IgG2a, IgG2b, IgG2c, and total IgG in plasma or B cell culture supernatant were measured using ELISA kits (Cat. No. 88-550450-22, eBioscience) according to the manufacturer’s instructions.

### Macrophage and T cell isolation

Bone marrow-derived macrophages were prepared as previously described^44^. Macrophage maturation was examined by flow cytometry with antibodies against F4/80 and CD11b (Supplementary Figure S21A). CD4+ or CD8+ T cells were isolated by using anti-mouse CD4 (Cat. No. 551539) or CD8 (Cat. No. 557766) conjugated to magnetic particles (BD Biosciences), respectively. T cell purity was evaluated by flow cytometry with antibodies against CD4 (Cat. No. 53-0041-82, eBioscience) or CD8 (Cat. No. 17-0081-82, eBioscience) (Supplementary Figure S21B and C).

### Splenocyte Activation Assay

Isolated splenic WT or miR-150KO B cells were activated for 72-hours with 10 μg/mL LPS, 10 ng/mL IL4 or 10 μg/mL anti-CD40 (Cat. No. 16-0401-85, eBioscience). Splenic CD4+ and CD8+ T-cells were activated with anti-CD3 (Cat. No. 14-0032-85, eBioscience) and anti-CD28 (Cat. No. 14-02281-86, eBioscience). Alternatively, CD4+ and CD8+ T cells, B-cells, and splenic macrophages were activated with a 5 hr PMA/ionomycin cocktail (Cat. No. E13495-124, eBioscience) in the presence of a protein transport inhibitor (Cat. No. 13496-122, eBioscience) per the manufacturer’s instructions.

### Co-culture assay

Following 72-hour activation with 10 μg/mL LPS, 10 ng/mL IL4 or 10 μg/mL anti-CD40, WT or miR-150KO B cells were mixed with WT CD4+ or CD8+ T cells at a ratio of 1:10 in the presence of anti CD3 and CD28. Activated WT B cells or miR-150KO B cells were also co-cultured with WT M1 or M2 BMDMs at a ratio of 1:10 using a trans-well plate. The activation of BMDMs or T cells was examined by flow cytometry with antibodies against CD69, CD80, CD86, IL2 or IFNγ after 48 h co-culture.

### Macrophage polarization analysis

A macrophage polarization assay was carried out following the protocol described previously^44^. To induce proinflammatory M1 macrophages, BMDMs were stimulated with LPS (100 ng/mL). IL4 (20 ng/mL) was used to induce anti-inflammatory M2 macrophages. After 48 h of stimulation, BMDMs were examined by flow cytometry with antibodies against CD69 (Cat. No. 45-0691-82, eBioscience), CD80 (Cat. No. 12-0801-85, eBioscience), and CD86 (Cat. No. 17-0862-82, eBioscience).

### Flow cytometry analysis

Unless otherwise specified, we purchased antibodies from eBioscience. Visceral stromal cells and splenic cells were stained with fluorescence-tagged antibodies to detect cell lineages. B cell subtypes were detected with antibodies against CD19 (Cat. No. 17-0193-82), CD5 (Cat. No. 11-0051-82) and CD43 (Cat. No. 12-0431-82); T cells were detected with antibodies against CD3 (Cat. No. 12-0031-82), CD4 or CD8; myeloid cells were detected with antibodies against F4/80 (Cat. No. 53-4801-82), CD11b (Cat. No. 17-0112-83) and Gr-1 (Cat. No. 45-5931-80); macrophage subtypes were detected with antibodies against F4/80, CD11b, CD206 (Cat. No. 141706, Biolegend), CD11c (Cat. No. 12-0114-83). Macrophage activation was measured with antibodies against CD80, CD69, CD86, TNFα (Cat No. 12-7321-41), and IL1β (Cat No.11-7114-80). T cell activation was measured using antibodies against CD80, CD86, CD69, IFNγ (Cat. No. 25-7311-82), and IL2 (Cat. No. 12-7021-82). B cell activation was measured using antibodies against CD80, CD86, CD69, and MHC II (Cat. No. 56-5321-82). Data were analyzed using Flowjo software or Accuri C6 software (BD Biosciences) or Kaluza software (Beckman Coulter).

### Quantitative reverse transcriptase-polymerase chain reaction (qRT-PCR) analysis

Total RNA was extracted from adipose tissue B cells or BMDMs using the Trizol extraction protocol according to the manufacturer’s instructions. Gene expression analysis was performed using a iScript One-Step RT-PCR kit with SYBR Green (Bio-Rad) on Bio-Rad CFX384 (Bio-Rad). The data presented correspond to the mean of 2^−ΔΔCt^ from at least three independent experiments after being normalized to β-actin. A list of primers and their sequences is presented in Supplementary Figure S22.

### Bio-Plex protein expression assay

The concentrations of IL1β, TNFα, IL6, IL10, and chemokine (C-C motif) ligand 2 (CCL2) in plasma were determined using a Bio-Plex^TM^ Cytokine Assay (Cat No. M60-009RDPD, Bio-Rad). Concentrations of insulin in plasma were determined using the Bio-Plex Pro Mouse Diabetes Insulin set (Cat. No. 171-G7006M, Bio-Rad). The levels of total and phosphorylated JNK in mature adipocytes were determined using the Bio-Plex Cell Signaling Magnetic Assays (Cat. No. 171-V50003M, Bio-Rad). β-actin (Cat. No. 171-V60020M, Bio-Rad) was used as the internal control. These Bio-Plex assays were performed using the Bio-Plex MAGPIX^TM^ multiplex reader (Bio-Rad). Results were analyzed using Bio-Plex Data Pro^TM^ software (Bio-Rad).

### Immunohistochemistry

Tissues collected from HFD-fed mice were fixed and stained with antibodies against F4/80 (Cat. No. 14-4801-82, eBioscience), B220 (Cat. No. 14-0452-82, eBioscience), CD3 (Cat. No. 14-0032-82, eBioscience) to detect macrophages, B cells, and T cells. Immunoglobulin protein was used as the negative control. Images were captured using a Zeiss Stallion Dual Detector Imaging System with Intelligent Imaging Innovations Software (Carl Zeiss).

### Western blotting

After homogenization using the BeadBug^TM^ microtube homogenizer (Benchmark Scientific), total protein was extracted from VATs using lysis buffer (Cell Signaling Technology). Protein concentrations were determined using the Bradford assay. Proteins were separated on PROTEAN^®^ TGX Stain-Free^TM^ Precast Gel (Bio-Rad) and transferred onto a polyvinylidene fluoride (PVDF) membrane followed by detection using antibodies directed against total Akt (Cat. No. 9272, Cell Signaling Technology) and phosphorylated Akt (Cat. No. 9271, Cell Signaling Technology).

### Luciferase reporter assay

The luciferase reporter assay was carried out following the protocol described previously10). Briefly, the full 3′-untranslated region (3′-UTR) sequence of the predicted target gene or at least 250-bp flanking the predicted miR-150 binding site was cloned into the psiCheck2 Vector (Promega) downstream of the *Renilla* luciferase-coding region. The reporter constructs were cotransfected with miR-150 mimic oligonucleotides or negative control oligonucleotides into HEK293 cells. After 48 h co-transfection, the activities of *Renilla* luciferase were measured with Dual-Glo luciferase reporter system (Promega) and normalized to the internal control firefly luciferase activity. Repressive effects of miR-150 on target genes were plotted as the percentage repression in three biological repeats that each contained three technical repeats.

### Lentiviral shRNA assay

The pLKO.1-CMV-TurboGFP^TM^ vector (Sigma-Aldrich) with inserted shRNA (targeting *Myb*, *Elk1* or *Etf1*) was co-transfected with compatible packaging plasmids into HEK293T cells. The lentiviral supernatants were collected after 72 h transfection and used to infect purified splenic miR-150 null B220+ B cells. Control vector with scramble target sites was used as the control.

### Data and statistical analyses

Results are expressed as means ± SEM. Each data point derived from qRT-PCR assays represents an average of two technical replicates, and data were averaged over independently replicated experiments (n = 3–4 independently collected samples) and analyzed using the Student’s *t* test. The overall group-effect was analyzed for significance using two-way ANOVA and Bonferroni post-test for each factor at each individual time. Data analyses were performed using Graphpad Prism version 6.0 software. A value of *P* < 0.05 was considered statistically significant.

### Study approval

All study protocols were reviewed and approved by the Institutional Animal Care and Use Committee of Texas A&M University. Methods were carried out in accordance with relevant and approved guidelines and regulations.

## Additional Information

**How to cite this article**: Ying, W. *et al.* miR-150 regulates obesity-associated insulin resistance by controlling B cell functions. *Sci. Rep.* **6**, 20176; doi: 10.1038/srep20176 (2016).

## Supplementary Material

Supplementary Information
