# Supplementary material for: miR-150 regulates obesity-associated insulin resistance by controlling B cell functions
Source: Sci Rep. 2016 Feb 1;6:20176. doi: 10.1038/srep20176 (PMC4735333; doi:10.1038/srep20176)
Supplement: Supplementary Information [file srep20176-s1.pdf]

Supplementary Figures

miR-150 regulates obesity-associated insulin resistance by controlling B cell functions

Wei Ying, Alexander Tseng, Richard Cheng-An Chang, Haiqing Wang, Yu-lieh Lin, Srikanth Kanameni, Tyler Brehm, Andrew Morin, Benjamin Jones, Taylor Splawn, Michael Criscitiello, Michael C. Golding, Fuller W. Bazer, Stephen Safe, Beiyan Zhou

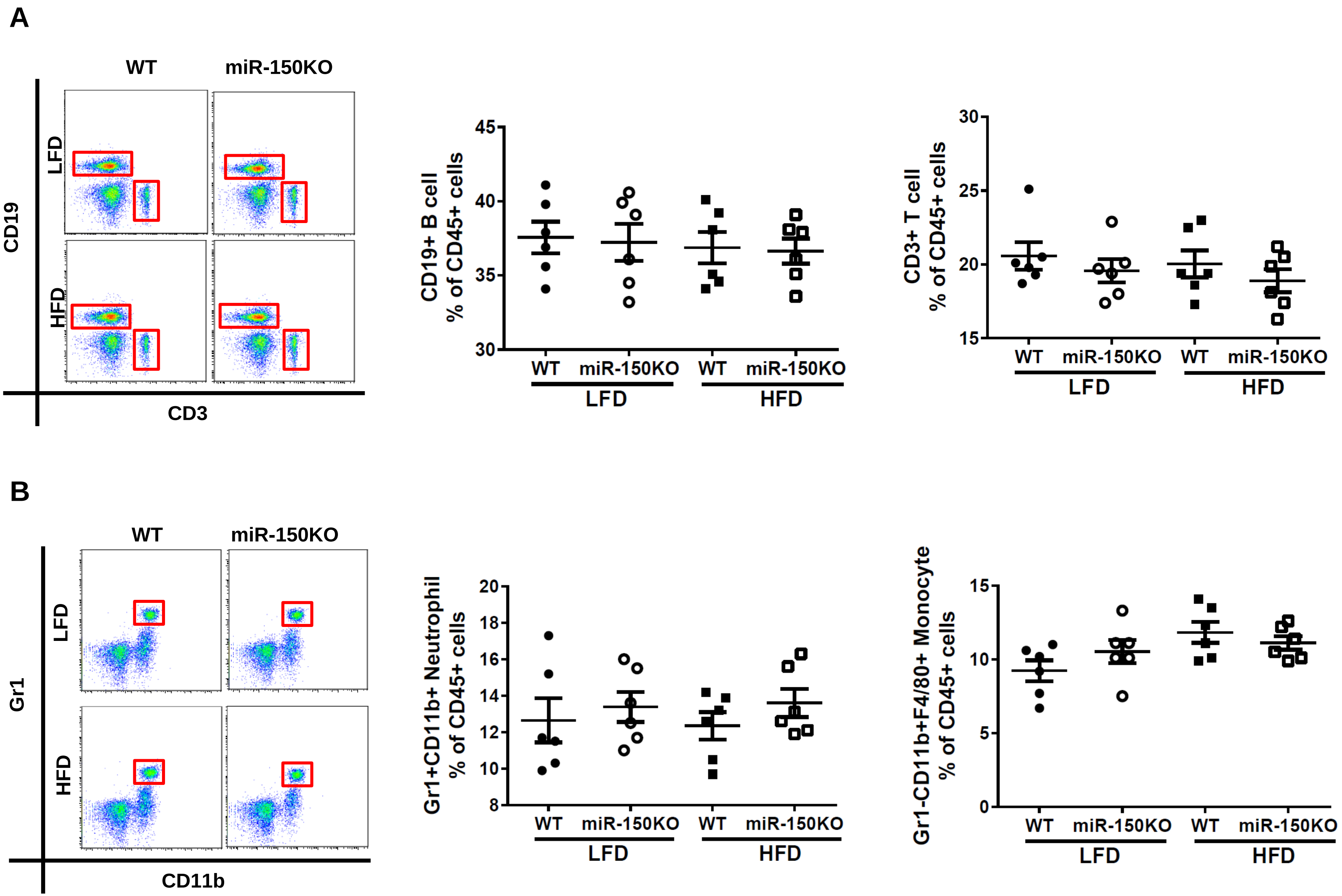

Supplementary Figure S1. Immune cell components in circulation of lean or obese mice. The population of CD19+ B cells (A), CD3+ T cells (A), neutrophils (B; Gr1+CD11b+), monocytes (B; Gr1-CD11b+F4/80+) in peripheral blood of wild type or miR-150KO mice with or without HFD. Data are presented as mean ± SEM. n=6.

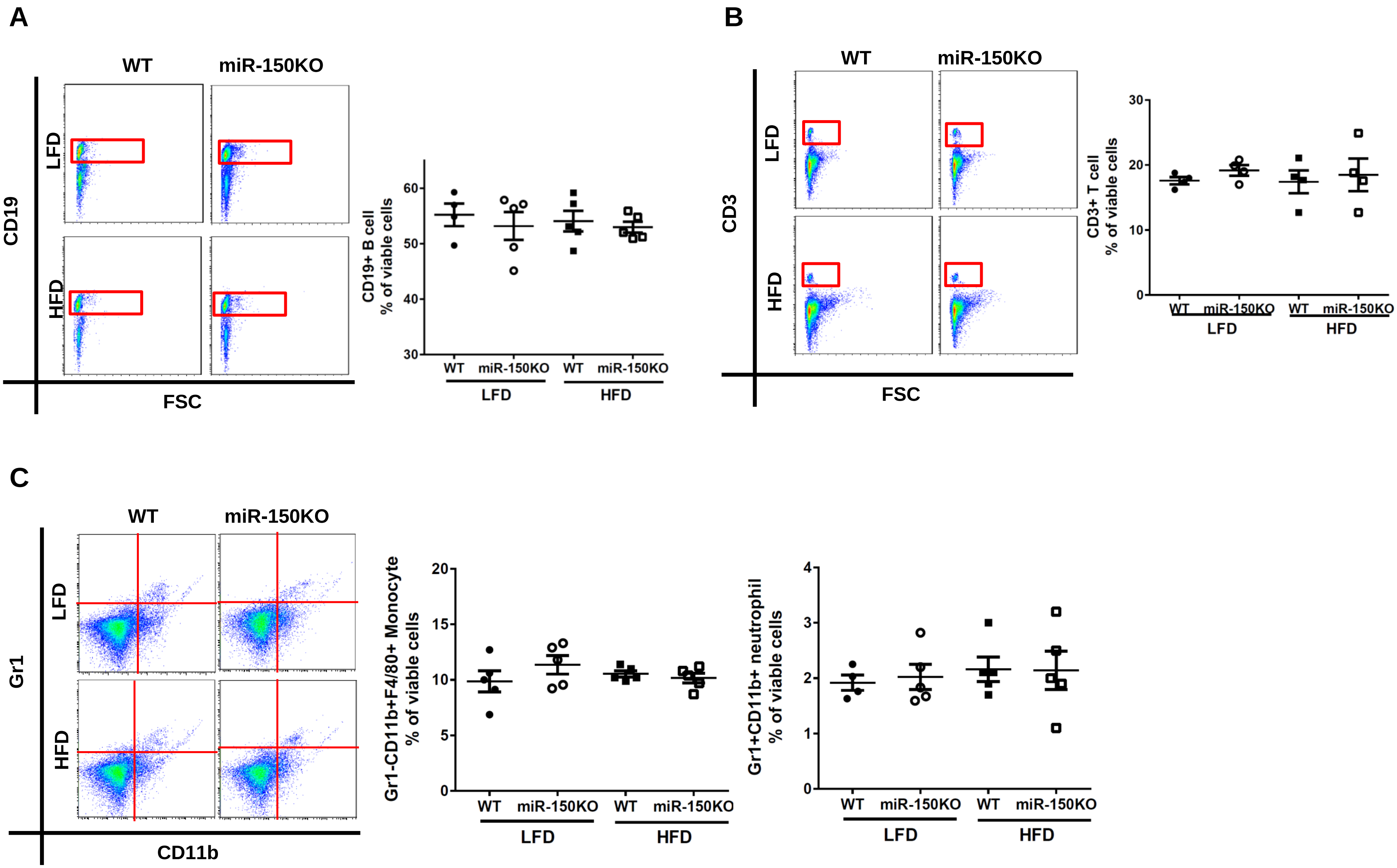

Supplementary Figure S2. Immune cell components in spleen of lean or obese mice. The population of CD19+ B cells (A), CD3+ T cells (B), neutrophils (C; Gr1+CD11b+), monocytes (C; Gr1-CD11b+F4/80+) in spleen of wild type or miR-150KO mice with or without HFD. Data are presented as mean ± SEM. n=6-8.

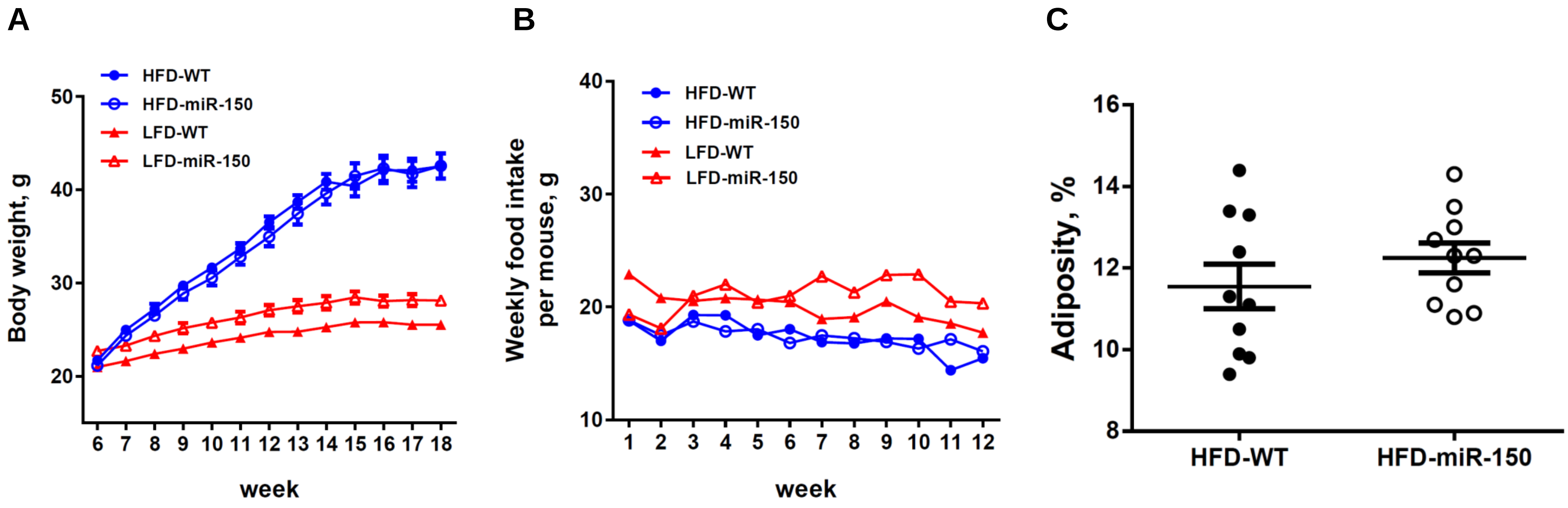

Supplementary Figure S3. Effects of miR-150 on growth performance of wild type (WT) or miR-150 null mice fed on low-fat diet (LFD) or high-fat diet (HFD). Body weight (A), food intake (B) and adiposity as measured by body fat percentage by weight (C) of mice were measured during 12-week feeding. Data are presented as mean ± SEM. n=10.

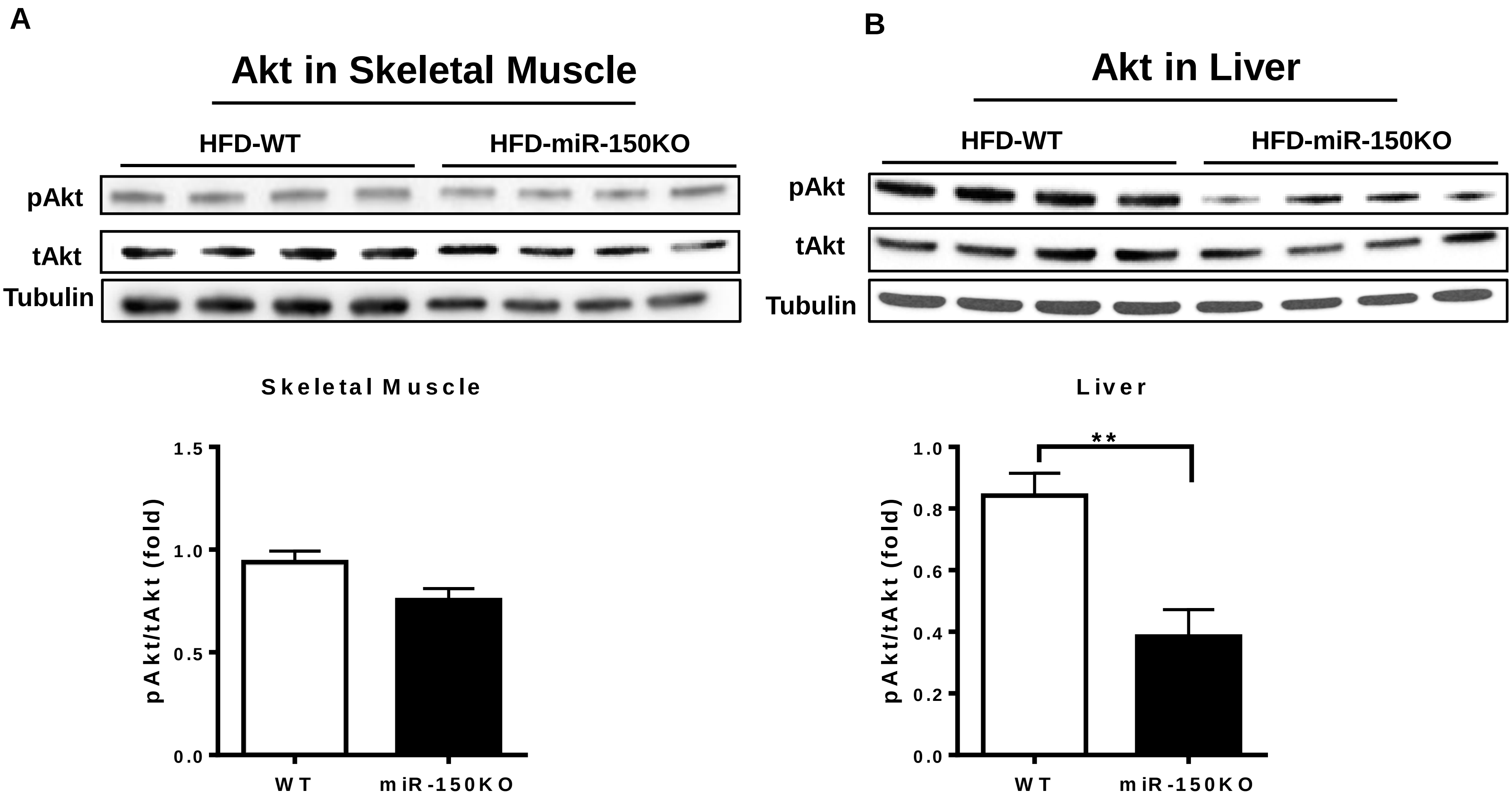

Supplementary Figure 4. miR-150KO mice demonstrate exacerbated insulin resistance. A and B, Activation of Akt signaling pathway in skeletal muscle and liver of obese WT and miR-150KO mice following portal vein insulin injection. Data are presented as mean ± SEM. n=4.

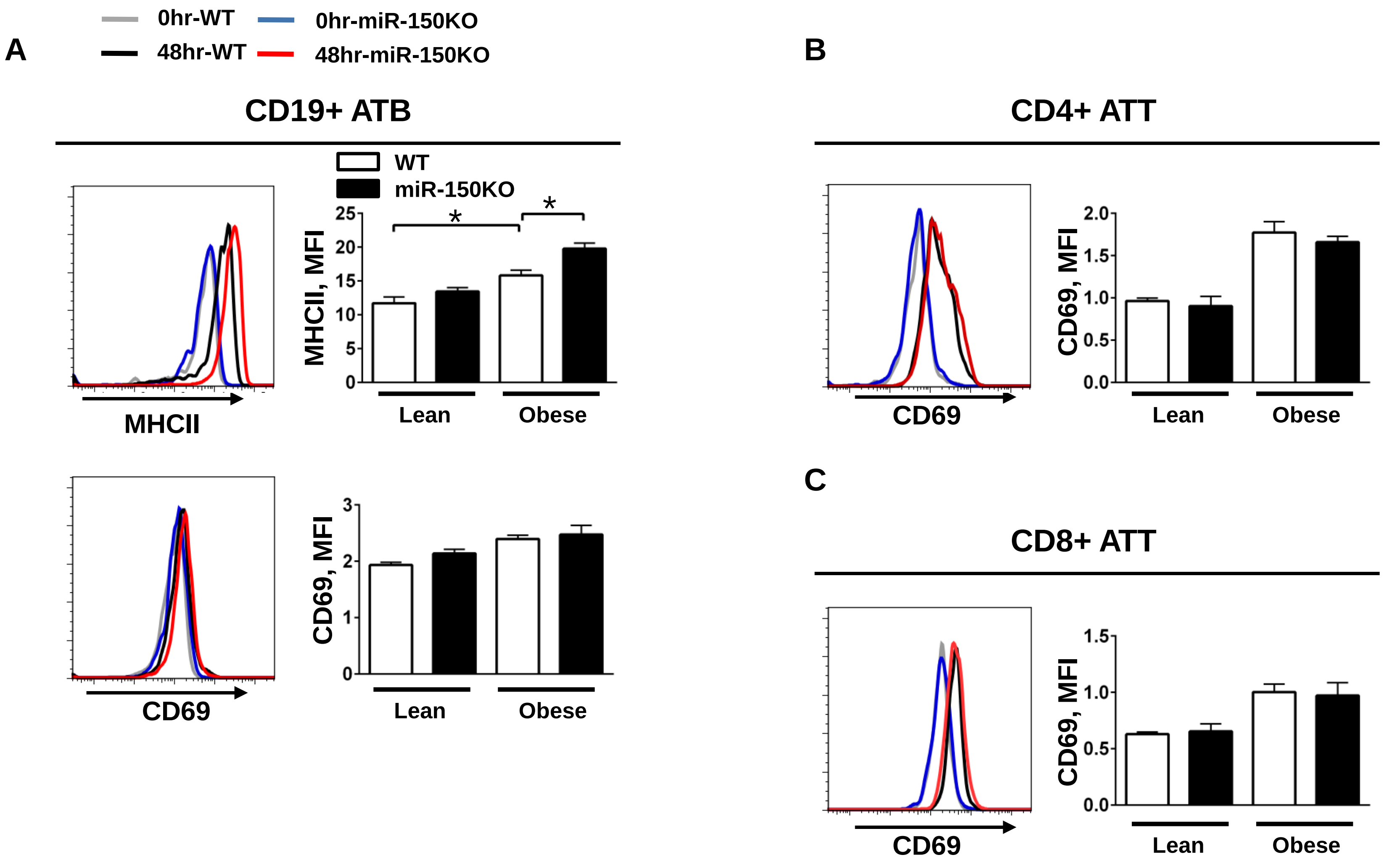

Supplementary Figure S5. Effects of miR-150 in adipose tissue immune cell activation. The expression of activation-related cell surface markers were measured in lean and obese WT and miR-150KO in VAT CD19+ B Cells (A), CD4+ T cells, and CD8+ T Cells (B and C). Data are presented as mean ± SEM. n=4.

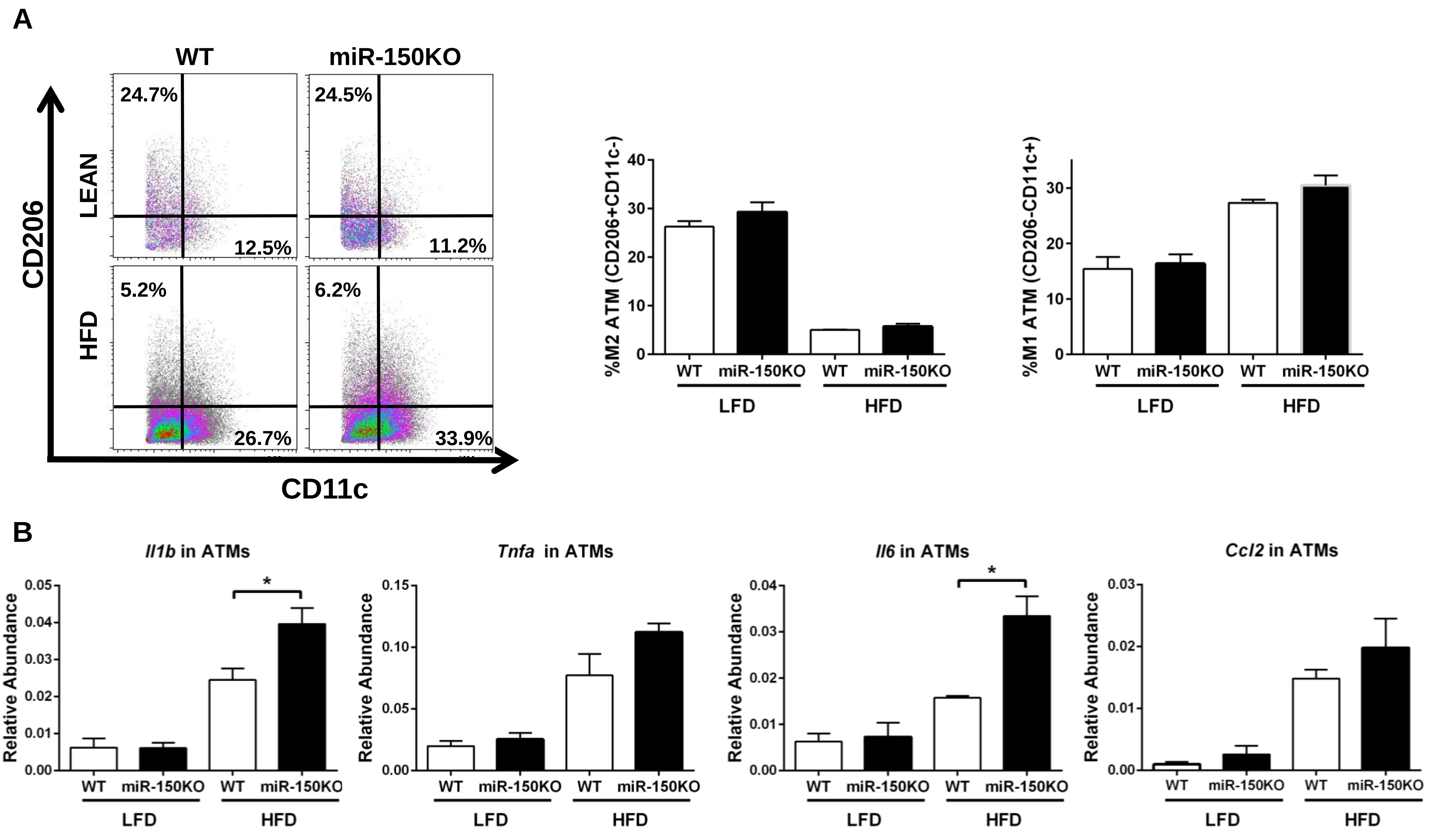

Supplementary Figure S6. ATMs from obese miR-150KO mice have an increased proinflammatory phenotype. Quantification of proinflammatory M1 and anti-inflammatory M2 macrophage cell proportions (A) and sorted ATM gene expression profiles (B) from lean and obese WT and miR-150KO mice. Data are presented as mean ± SEM. n=4.

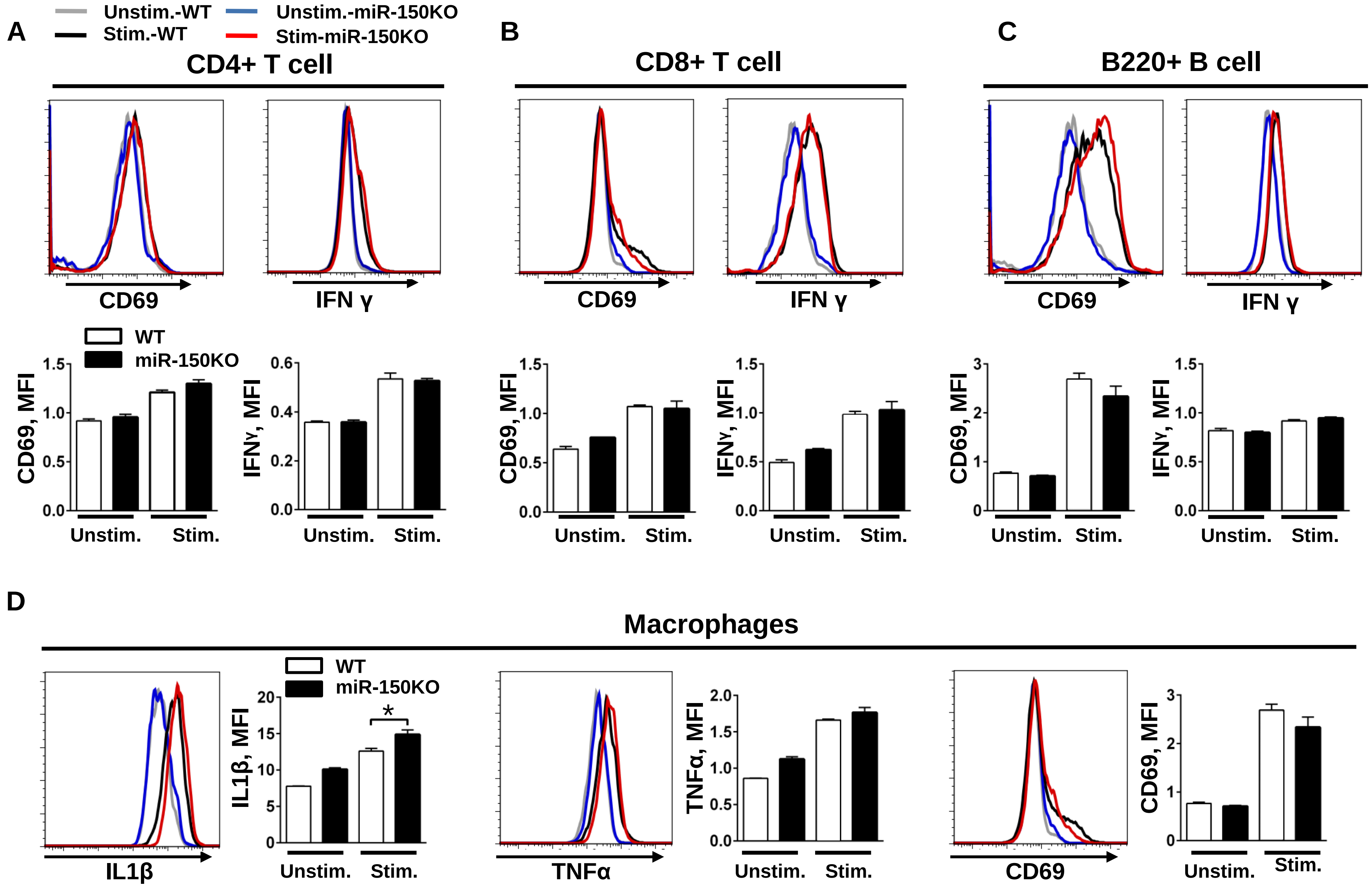

Supplementary Figure S7. Effects of miR-150 on acute immune cell activation. Splenic CD4+ (A) or CD8+ T cell (B), B220+ B cell (C), and macrophage (D) activation markers and cytokine production were measured after 5 hours of PMA/ionomycin stimulation. Data are presented as mean ± SEM. n=4.

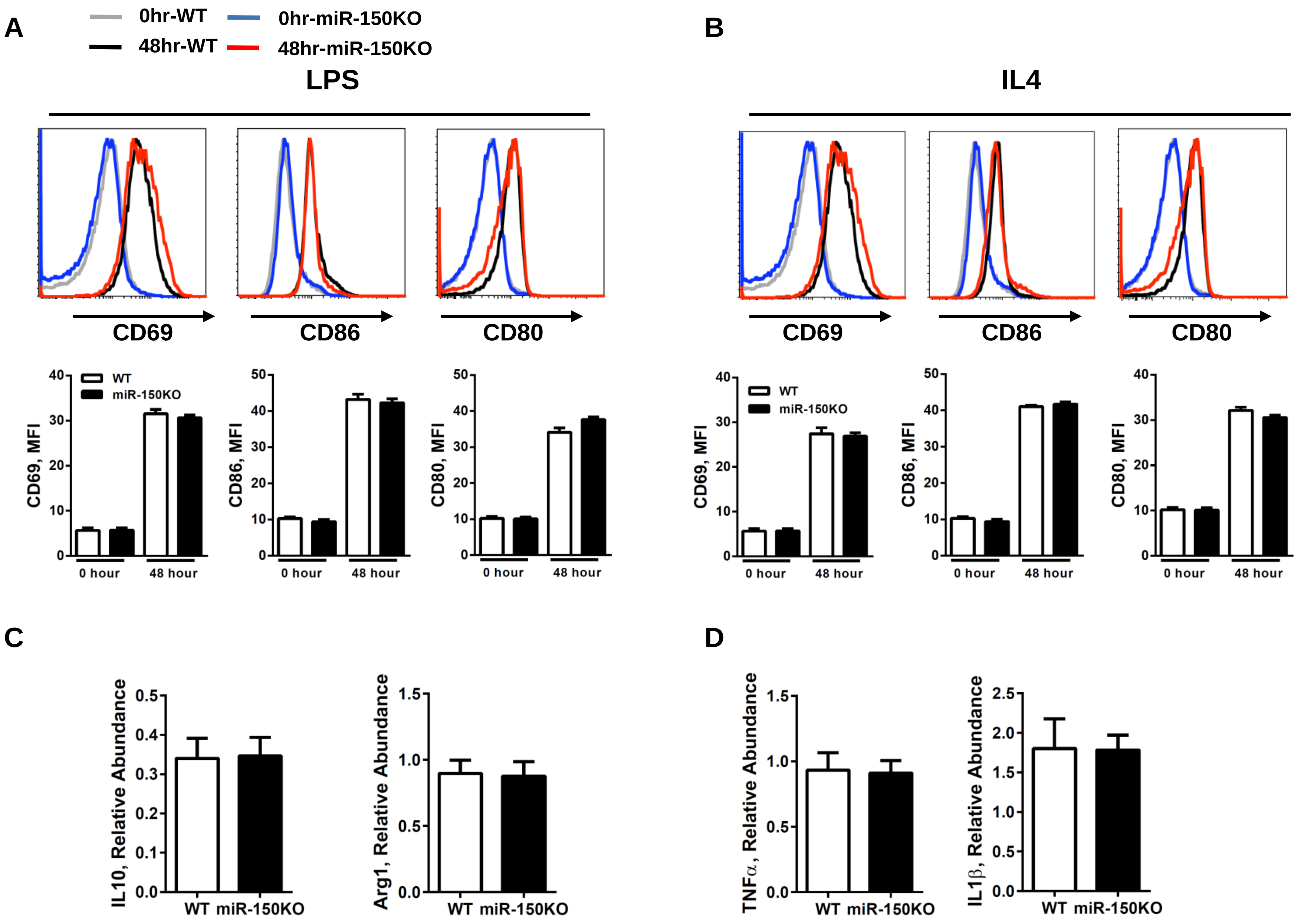

Supplementary Figure S8. Effects of miR-150 on macrophage activation. The expression of activation-related cell surface markers (A and B) and key genes (C and D) were measured after 48 hours either LPS or IL4 stimulation. Data are presented as mean ± SEM. n=3.

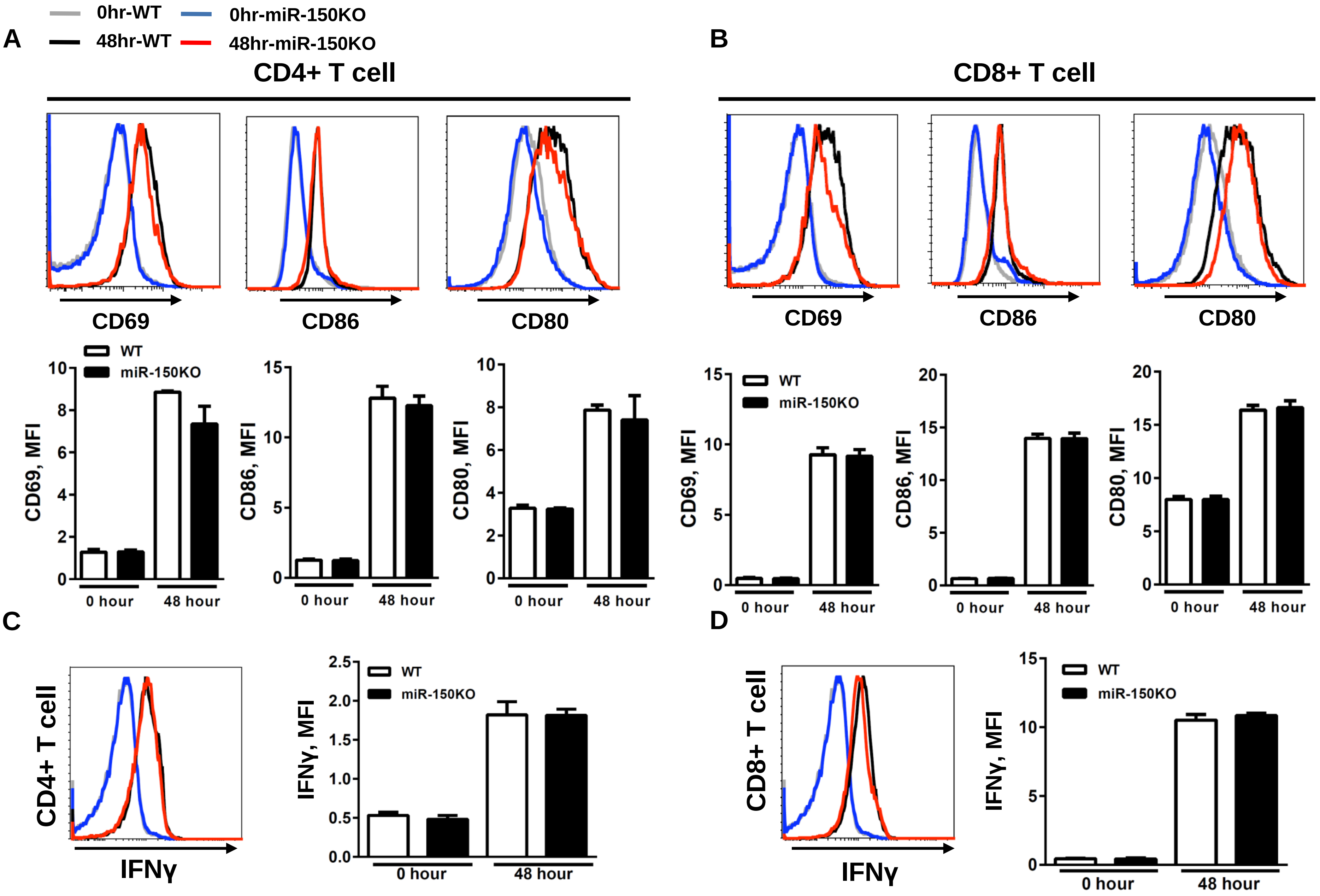

Supplementary Figure S9. Effects of miR-150 on activation of CD4+ or CD8+ T cell. The expression of activation-related cell surface markers (A and B) and key genes (C and D) were measured after 48 hours anti-CD3/CD28 stimulation. Data are presented as mean ± SEM. n=3.

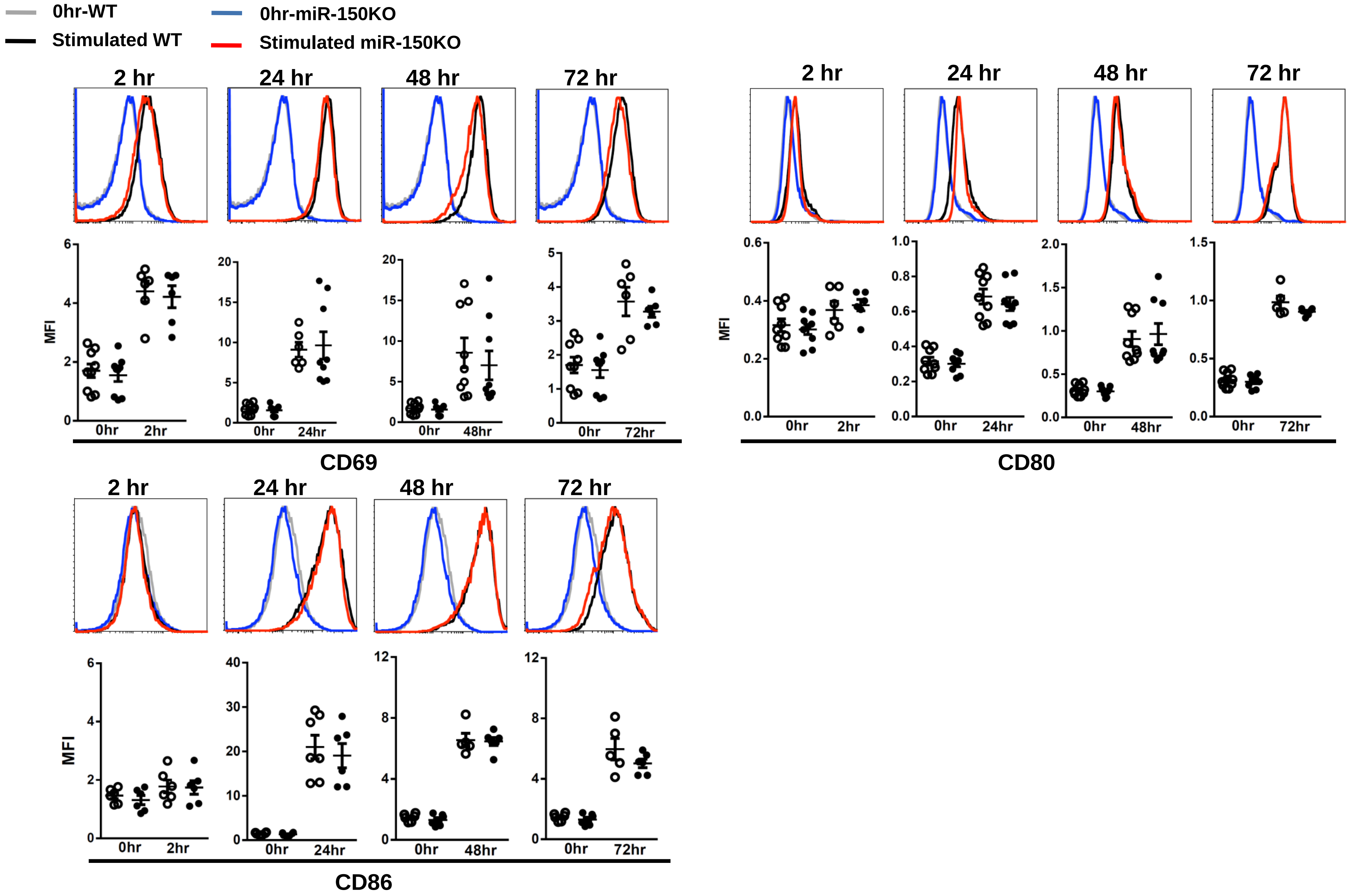

Supplementary Figure S10. Effect of miR-150 on the expression of activation-related cell surface markers of B cell activation. The expression of activation-related cell surface markers, CD69, CD80, and CD86, were monitored during IL4/CD40-stimulated B cell activation. Data are presented as mean ± SEM. n=6-8.

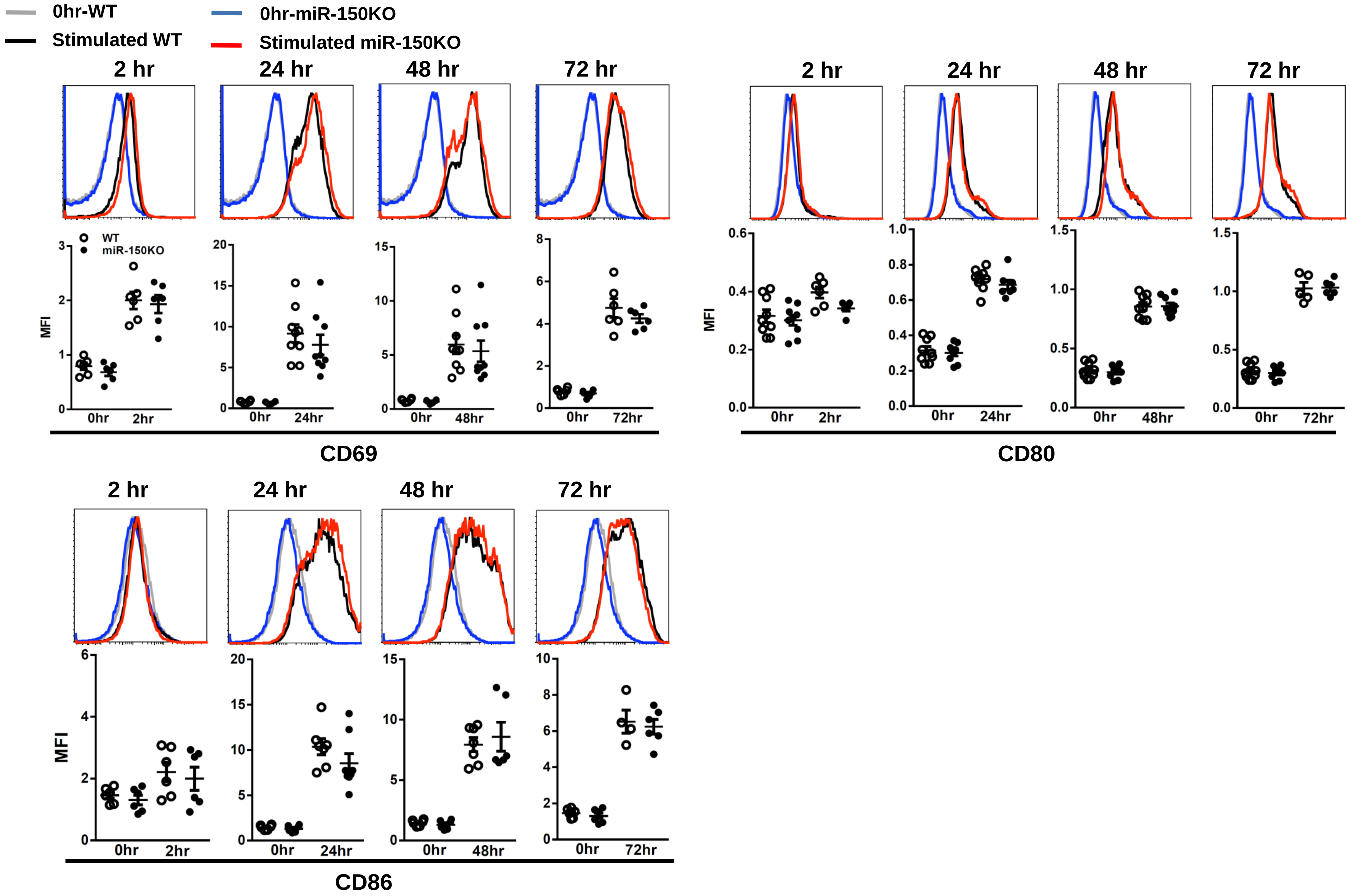

Supplementary Figure S11. Effect of miR-150 on the expression of activation-related cell surface markers on B cells. The expression of activation-related cell surface markers, CD69, CD80, and CD86, were monitored during LPS-stimulated B cell activation. Data are presented as mean ± SEM. n=6-8.

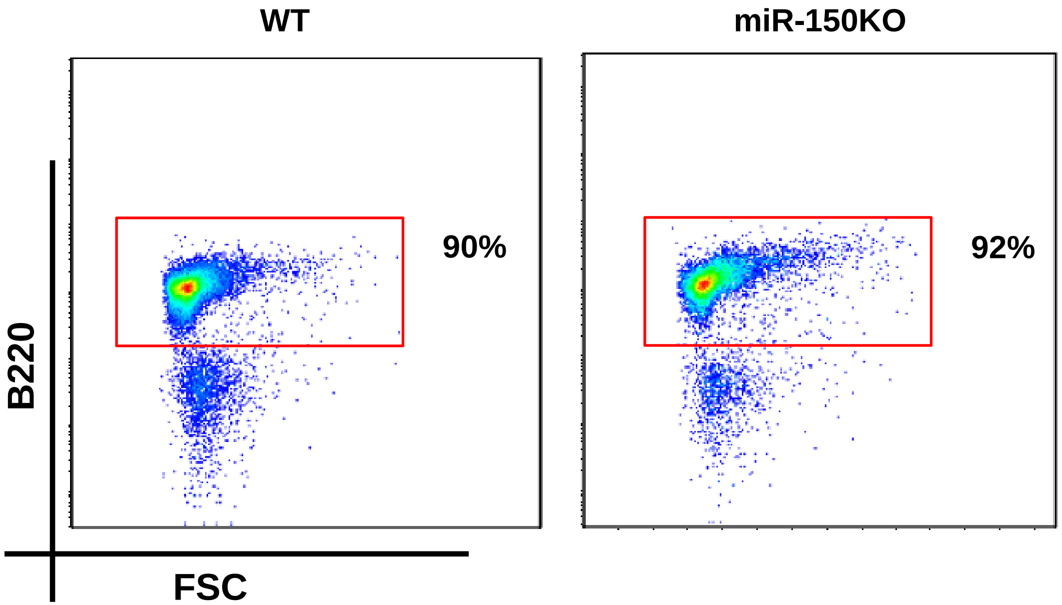

Supplementary Figure S12. Validation of B cell purity. B220+ B cells were isolated from HFD-fed mice spleens with anti-mouse B220 conjugated magnetic beads. The purity of B220+ cells were evaluated by flow cytometry analysis with antibody against B220.

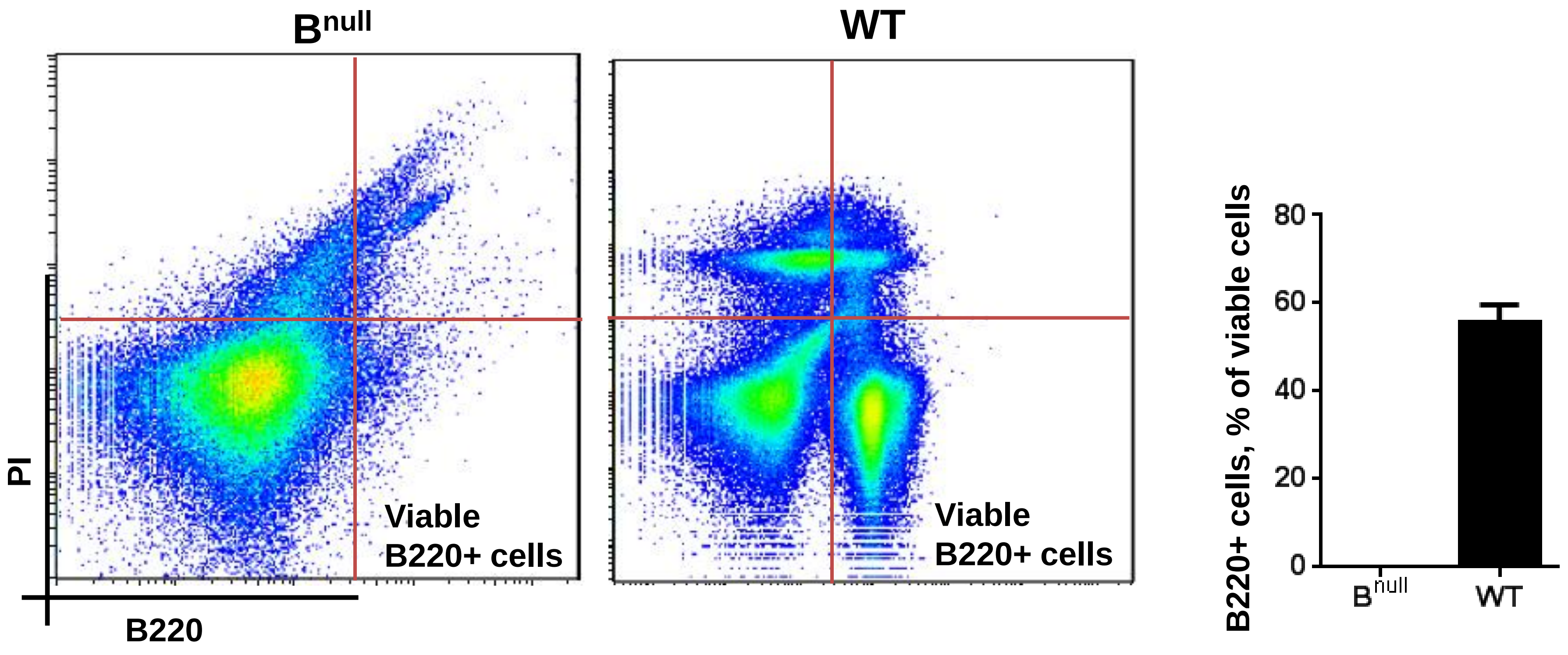

Supplementary Figure S13. Mature B cell deficiency in B^{null} mice. Viable CD19^{+} B cells in the spleen of C57BL/6 immunoglobulin μ heavy-chain knockout (B^{null}) mice or wild type (WT) mice were analyzed by flow cytometry with antibodies against B220; n=6. Data are presented as mean ± SEM.

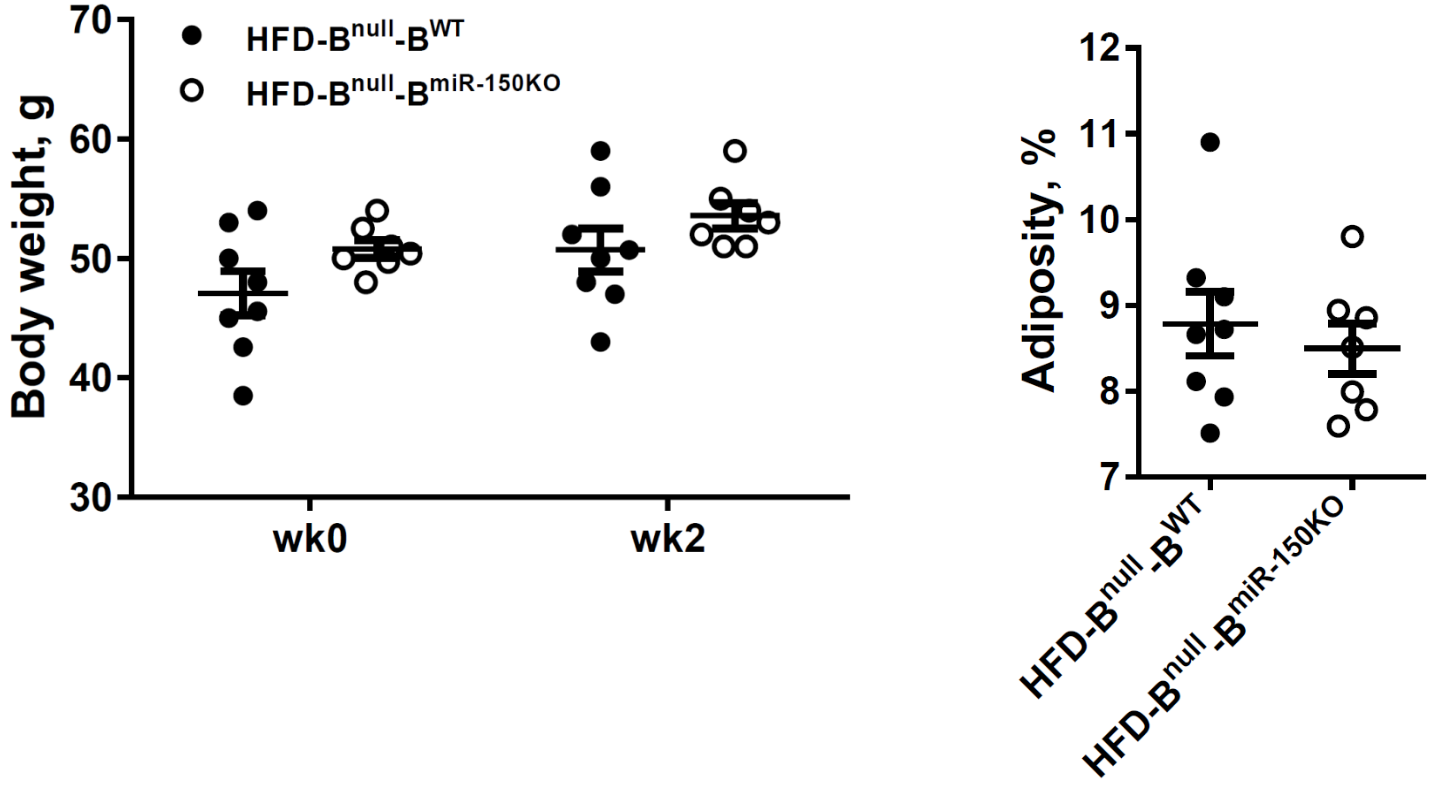

Supplementary Figure S14. Body weight and adiposity of HFD-fed B^{null} mice after received WT B cells or miR-150KO B cells. Data are presented as mean ± SEM. n=8.

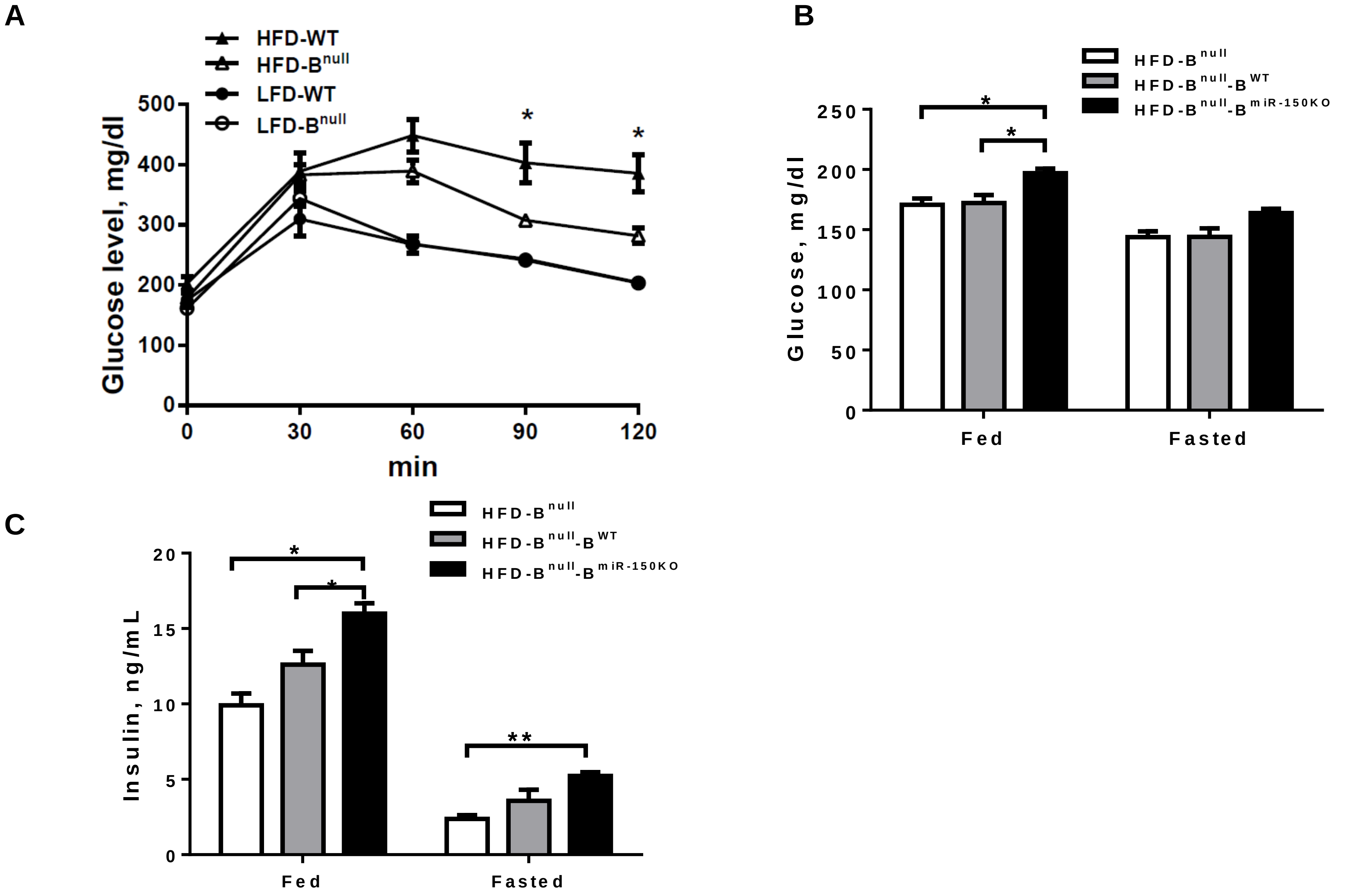

Supplementary Figure S15. Glucose and insulin levels of B^{null} mice after WT or miR-150 KO B cells infusion. Glucose tolerance test of B^{null} or WT mice after 12 weeks LFD or HFD feeding (A). Fed and fasted glucose (B) or insulin (C) levels after WT or miR-150KO B cell infusion. Data are presented as mean ± SEM. n=5-8

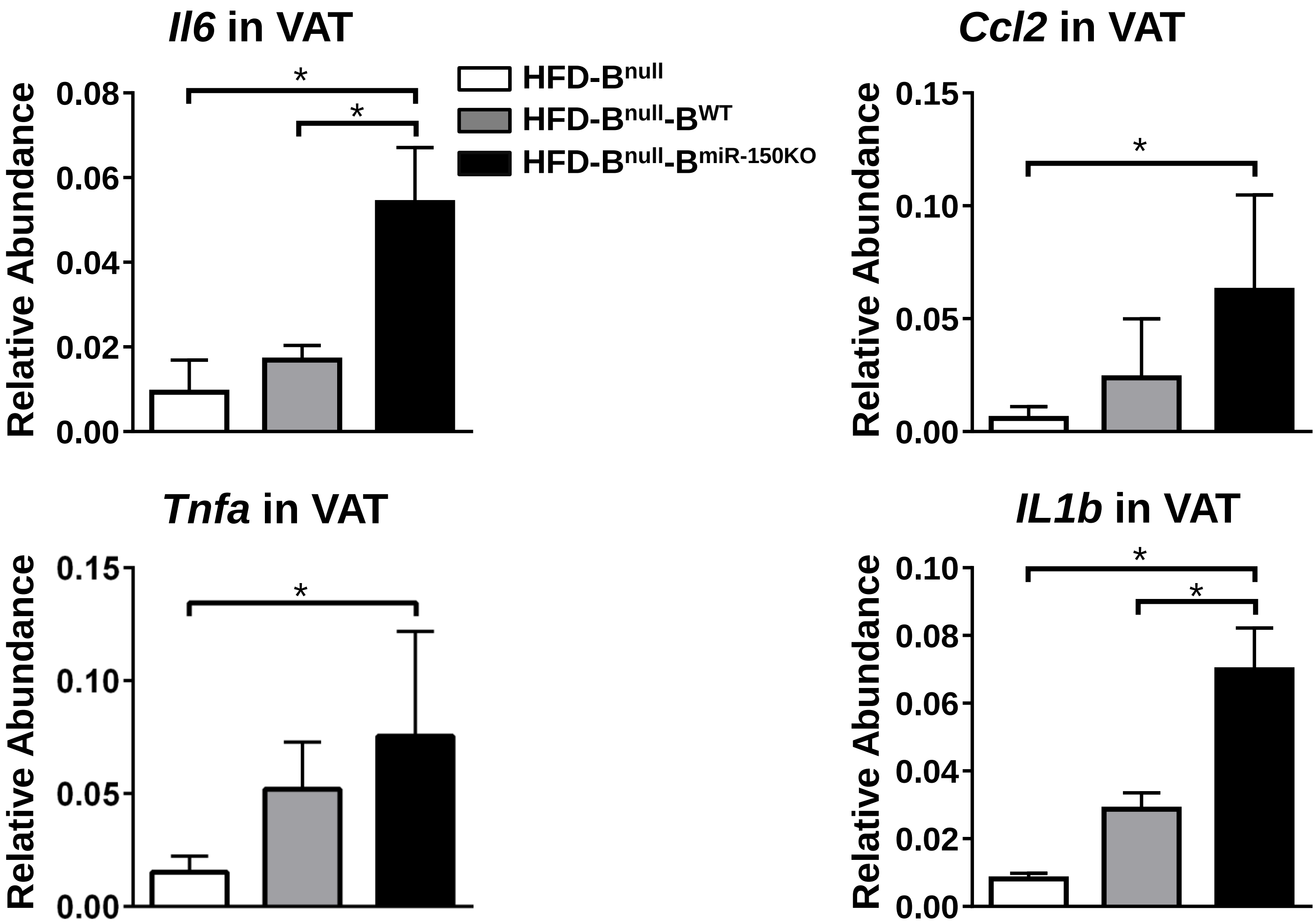

Supplementary Figure 16 Inflammatory gene expression in VAT of HFD-fed B^{null} mice after receiving WT B cells or miR-150KO B cells. Data are presented as mean ± SEM. n=8.

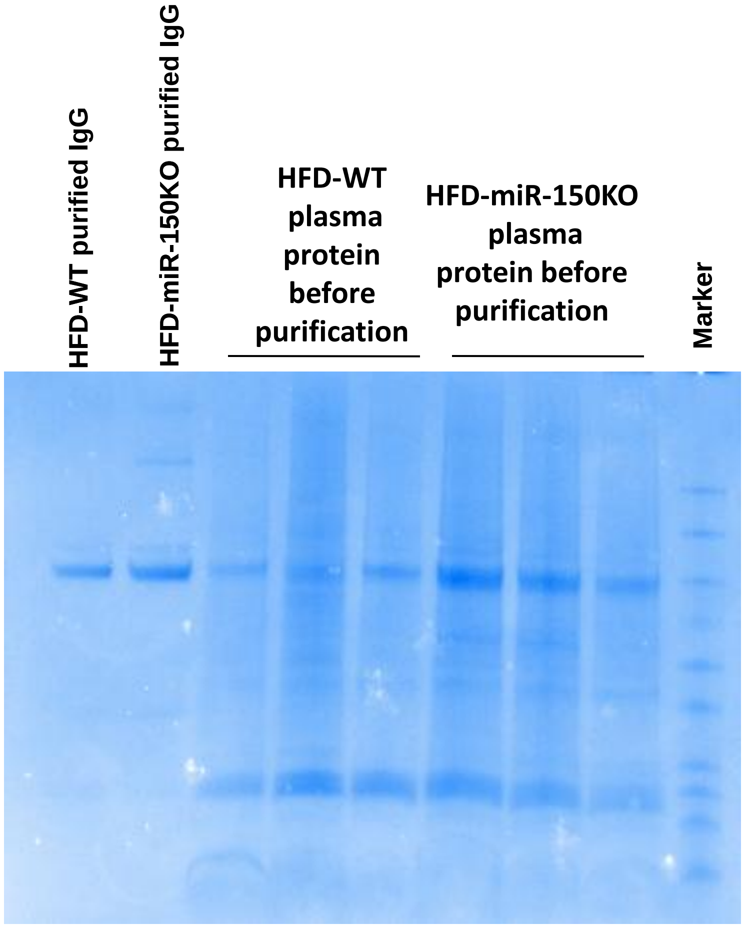

Supplementary Figure S17. Validation of immunoglobulin purification. Immunoglobulin proteins were purified from plasma of HFD-fed WT mice or HFD-fed miR-150KO mice.

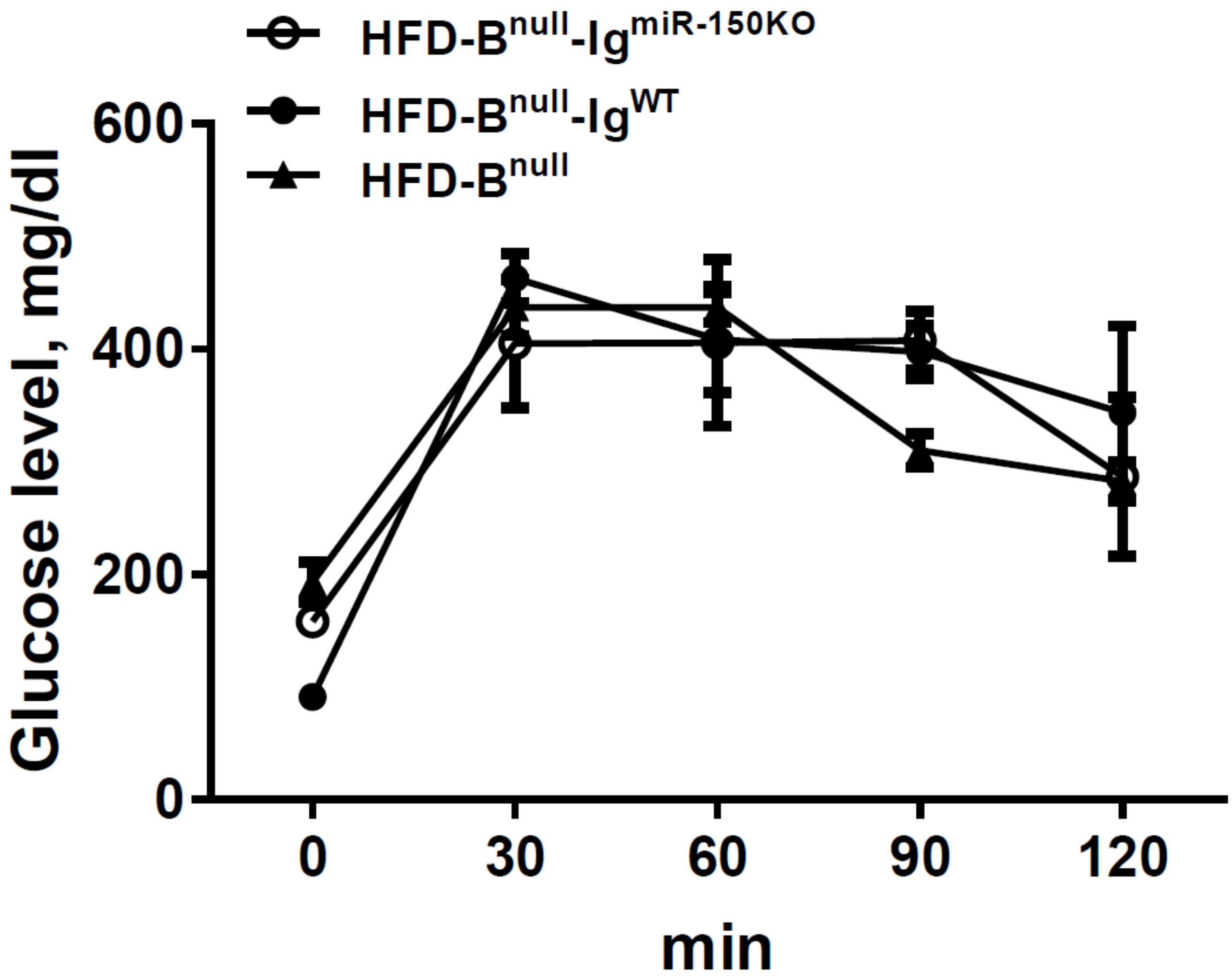

Supplementary Figure S18. Glucose tolerance test of HFD-fed B^{null} mice after received purified immunoglobulins. HFD-B^{null}-IgG$^{miR-150KO}$, HFD-fed B^{null} mice with immunoglobulin (Ig) purified from HFD-fed miR-150KO mice, HFD-B^{null}-IgG^{WT}: HFD-fed WT mice. Data are presented as mean ± SEM. n=6

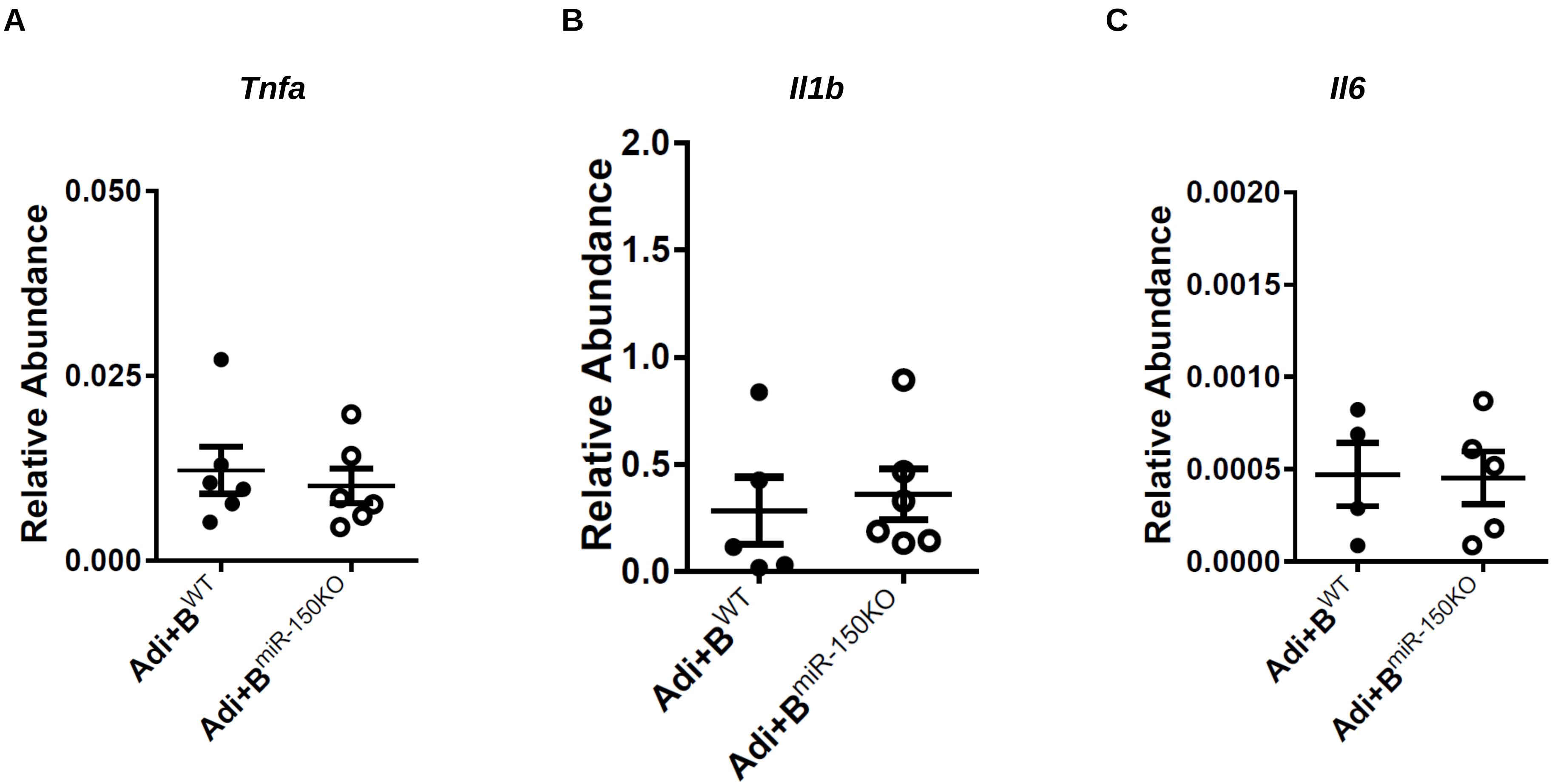

Supplementary Figure S19. Effect of miR-150 on the interaction between B cell and adipocytes. The expression of proinflammatory cytokines *Tnfa* (A), *Il1b* (B), and *Il6* (C) by adipocytes (Adi) after 48 hours co-cultured with either activated wild type B cells (B^{WT}) or miR-150KO B cells ($B^{miR\text{-}150KO}$). Data are presented as mean ± SEM. n=4-6.

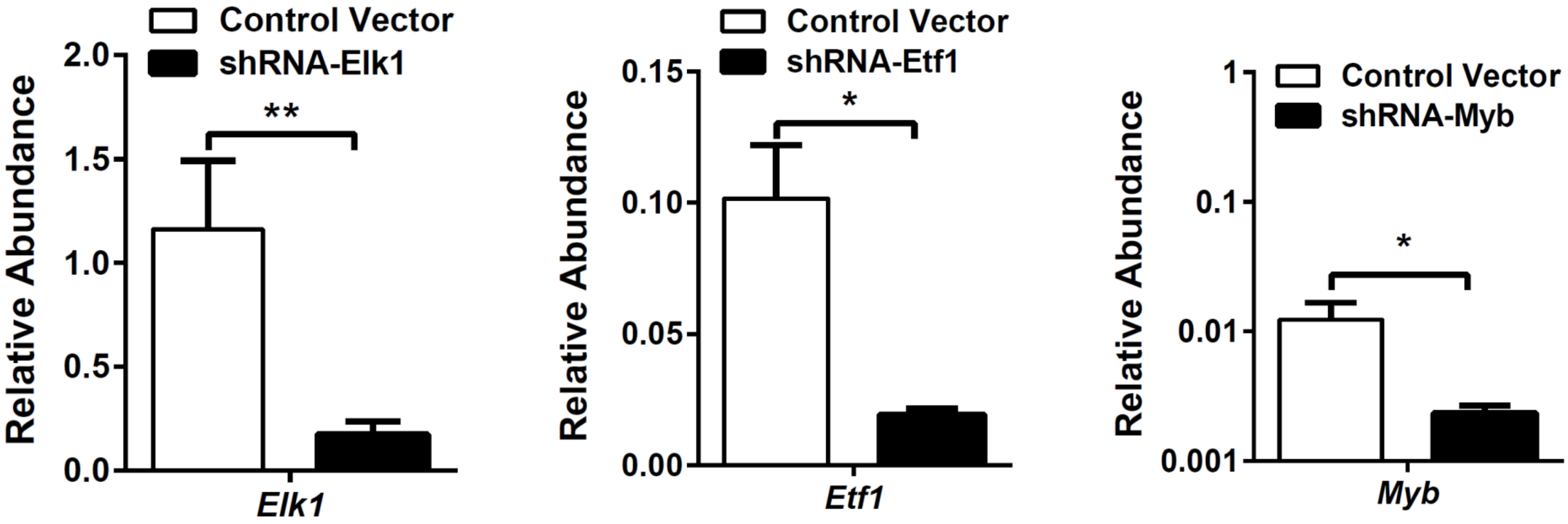

Supplementary Figure S20. Knockdown of miR-150 target gene expression in miR-150KO B cells by short hairpin RNA (shRNA) assay. Data are presented as mean ± SEM. n=3.

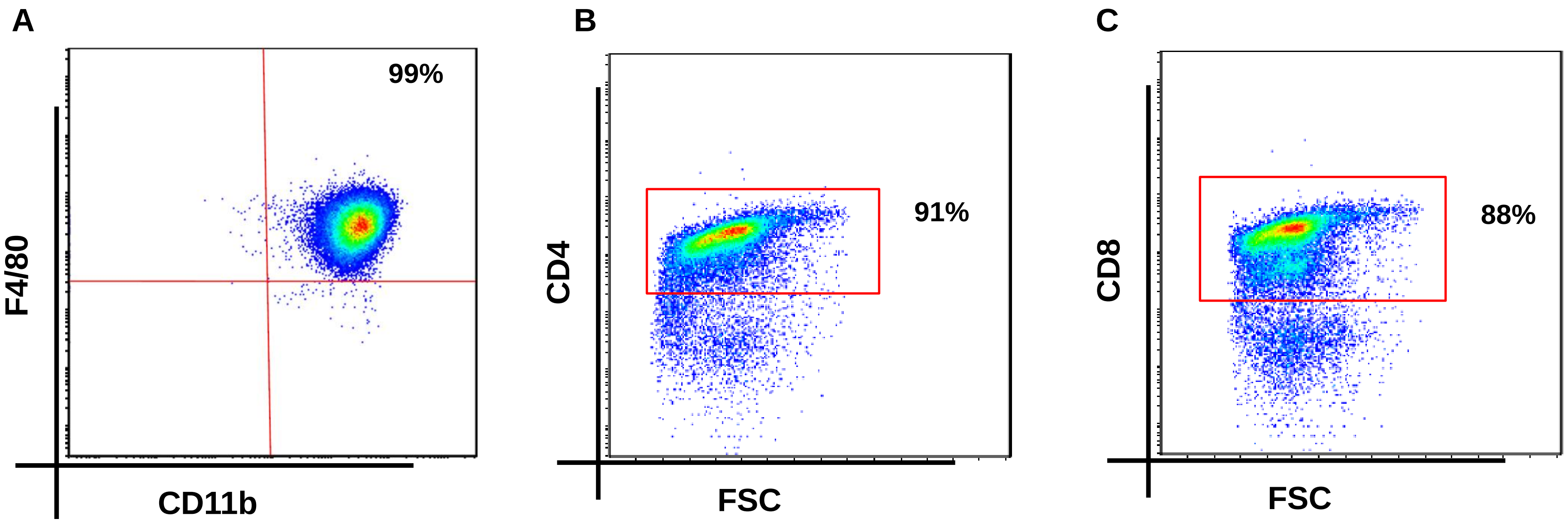

Supplementary Figure S21. Cell isolation. Bone marrow-derived macrophages (a), purified splenic CD4+ T cells (b), and purified splenic CD8+ T cells (c), were analyzed by flow cytometry with antibodies against F4/80, CD11b, CD4, and CD8, respectively.

Primer	Forward	Reverse
TNFa	5'-TTGTCTACTCCCAGGTTCTCT-3'	5'-GAGGTTGACTTTCTCCTGGTATG-3'
IL1b	5'-TGGACCTTCCAGGATGAGGACA-3'	5'-GTTCATCTCGGAGCCTGTAGTG-3'
Btk	5'-GTCAGAGACTCCAGCAAAGCTG-3'	5'-CCAGGTAATACTGGCTCTGTGG-3'
Blnk	5'-AGATGGACCGTTCCACAGCTTC-3'	5'-CTCTTCAGCAGACTTGCGGTCA-3'
Mcp1	5'-ATGCAGGTCTCTGTCACG-3'	5'-AGTATGACAGAGAACTAG-3'
Myb	5'-TGAGAAGCTGAAGAAGCTGGTGGA-3'	5’-TGACTTTCCAGTCGTCTGTTCCGT-3'
Arg1	5'-CTCCAAGCCAAAGTCCTTAGAG-3'	5'-AGGAGCTGTCATTAGGGACATC-3'
Elk1	5'-AAGAATTGGAAGCTGCAAGGGCTG-3'	5'-ACACCTGGGCTGTGTTCTCTGTTA-3'
Etf1	5'-ATTTGGGAGCCCTCTGTGCCATTA-3'	5'-TAGCACAGAAGCCCATCATGTCCA-3'
Lyn	5'-TCCTCAAGAGTGATGAAGGTGGC-3'	5'-ACGGTGGATGATGTTCTTCCGC-3'
Il6	5'-CAAGAGACTTCCAGCCAGTTGC-3'	5'-TTGCCGAGTAGACCTCATAGTGACC-3'
IL10	5'-CGGGAAGACAATAACTGCACCC-3'	5'-CGGTTAGCAGTATGTTGTCCAGC-3'
Src	5'-GTTGCTTCGGAGAGGTGTGGAT-3'	5'-CACCAGTTTCTCGTGCCTCAGT-3'
Syk	5'-GAGAGCACTGTGTCCTTCAACC-3'	5'-CAGCATAAGGGCTCTGCTACAC-3'
B-Actin	5’-GCCACCGATCCACACAGACT-3’	5’-GCTCTGGCTCCTAGCACCAT-3’

Supplementary Figure S22. List of primers and sequences used for qRT-PCR
